# Plasma lipidomics, choline metabolites, and metabolic-associated steatotic liver disease (MASLD): A Coronary Artery Risk Development in Young Adults (CARDIA) study

**DOI:** 10.1371/journal.pone.0341462

**Published:** 2026-03-13

**Authors:** Jessica K. Sprinkles, Qiyao Qin, Charles Steward, Annie Green Howard, Anju Lulla, Autumn G. Hullings, J Jeffrey Carr, Saame Raza Shaikh, Christy L. Avery, Kari E. North, Penny Gordon-Larsen, Katie A. Meyer

**Affiliations:** 1 Department of Nutrition, Gillings School of Global Public Health, University of North Carolina at Chapel Hill, Chapel Hill, North Carolina, United States of America; 2 Nutrition Research Institute, University of North Carolina at Chapel Hill, Kannapolis, North Carolina, United States of America; 3 Department of Biostatistics, Gillings School of Global Public Health, University of North Carolina at Chapel Hill, Chapel Hill, North Carolina, United States of America; 4 Carolina Population Center, University of North Carolina at Chapel Hill, Chapel Hill, North Carolina, United States of America; 5 Department of Radiology and Radiological Sciences, Vanderbilt University Medical Center, Nashville, Tennessee, United States of America; 6 Department of Epidemiology, Gillings School of Global Public Health, University of North Carolina at Chapel Hill, Chapel Hill, North Carolina, United States of America; Universita degli Studi della Campania Luigi Vanvitelli Scuola di Medicina e Chirurgia, ITALY

## Abstract

Metabolic-associated steatotic liver disease (MASLD) is marked by accumulation of hepatic triacylglycerols (TAG), but many other lipids have been implicated. Choline metabolism has been shown to be related to MASLD, specifically through phosphatidylcholines (PC) role in hepatic TAG removal through very low density lipoproteins (VLDL). There are a lack of population-based studies with integrated data on lipidomics, choline metabolites, and MASLD. We tested associations between the plasma lipidome, choline metabolites, and MASLD using data from the Coronary Artery Risk Development in Young Adults (CARDIA) Study. The analytic sample included 1,039 participants with data on choline metabolite, lipidomic, and liver attenuation data [mean (SD) age: 45 (4); 57% female; 57% White race]. MASLD (n = 234) was defined as mean CT-derived liver attenuation < 51HU. Plasma lipidomics and choline metabolites were quantified from stored fasting plasma using liquid-chromatography and infusion-mass spectrometry. In logistic regression adjusted for sociodemographics, lifestyle, and clinical variables, total TAGs, diacylglycerols (DAG), and dihydroceramides (DCER) were positively, and lactosylceramides (LCER) were inversely, associated with MASLD. Species-level results revealed diverging MASLD associations for PCs, based on FA composition. In choline metabolite models, betaine was inversely associated with MASLD. A lipidomic risk score (LRS) derived from penalized regression of MASLD on lipid species was associated positively with choline, and inversely with betaine. We contribute population-based results to a growing literature relating lipidomics and MASLD. In our data, FA composition is biologically relevant to MASLD, particularly for PCs and TAGs. Our results link choline metabolites to both the plasma lipidome and to incident MASLD, furthering efforts in biomarker development and supporting mechanistic evidence using population-level data.

## Introduction

The global incidence of non-alcoholic fatty liver disease (MASLD), defined as 5% or greater hepatic steatosis in people without heavy alcohol use or other causes of hepatic steatosis, has more than doubled over the past three decades [1,2]. People with MASLD have an increased risk of cirrhosis, liver failure, and hepatocellular carcinoma [3]. MASLD is primarily characterized by the accumulation of triacylglycerols (TAG) in the liver, known as intra-hepatic TAGs. Very low-density lipoproteins (VLDL) export TAGs from the liver, but when the influx of free fatty acids (FA) to the liver exceeds the outflux of TAG-VLDLs, intra-hepatic TAGs accumulate [3]. Phosphatidylcholine (PC), a phospholipid metabolite of dietary choline, is required for the assembly and secretion of these VLDLs [4]. Thus choline-deficient diets, which have long been used in mouse models of fatty liver, disrupt phosphatidylcholine and VLDL production to promote liver steatosis [2]. These mechanisms, along with the high prevalence of choline deficient diets in U.S. adults [5], warrant joint study of lipid and choline metabolism in MASLD.

Recent technological advancements in plasma lipidomics have allowed broad spectrum quantification and identification of functionally diverse molecular lipid species, bridging the gap between epidemiologic and mechanistic research [6]. Significant physiological differences exist across the lipid classes (denoted by the lipid head group) and lipid species (denoted by the FA tails). In general, shorter, more saturated fatty acids negatively associate with cardiometabolic measures, while longer, more unsaturated fatty acids positively associate with cardiometabolic measures, through inflammatory mechanisms [7]. Using lipidomic data at the species-level allows consideration of the length and saturation of FA tails, revealing lipid metabolism abnormalities in MASLD with greater detail than class-level analyses. MASLD has long been associated with increased TAGs and free FAs, and decreased HDL-c and PC, but many other lipids are implicated in the MASLD development. For example, diacylglycerols (DAG) and ceramides (CER) have roles in insulin signaling and ER stress [3]; lysophospholipids influence inflammation and insulin resistance [8]; and lipid species containing long-chain poly-unsaturated FAs reduce hepatic TAGs to benefit MASLD [9].

Despite the direct link through PC production, evidence linking choline- and lipid- metabolism in MASLD is limited.. PC is the primary metabolite of choline, but betaine may also impact MASLD through roles in methyl group supply, lipid metabolism regulation, insulin signaling, and anti-oxidant effects [10–13]. Inverse associations between betaine and liver disease have been reported in clinic-based and cross-sectional studies [14,15]. Previous lipidomic studies of MASLD have been mostly conducted in clinic-based [16–19] or small case-control samples [20–23]. The need for epidemiologic studies of the plasma lipidome and MASLD has been acknowledged in the literature, given that no biomarker for MASLD currently exists and histological assessment is required for diagnosis [21,24]. In addition, recent changes in terminology, from NAFLD to MASLD, and diagnostic criteria have added a level of complexity to interpretation of the literature. Studies indicate that roughly 99% of individuals classified as NAFLD will meet MASLD criteria, though this estimate is drawn largely from clinic-based samples [25–27]. We expect decreased comparability between NAFLD and MASLD in population-based data. To our knowledge, no population-based study has assessed the associations between the plasma lipidome, choline metabolites, and MASLD.

In this analysis, we leverage data from the Coronary Artery Disease Risk in Young Adults (CARDIA) Study to investigate relationships between the plasma lipidome, choline metabolites, and MASLD in a population-based study of early-middle aged U.S. adults of black and white race. Based on mechanistic understanding and previous human studies, we hypothesized positive associations between total TAGs, DAGs, and CERs with MASLD; and inverse associations between total PCs and MASLD. In addition, we hypothesized that lipid species containing shorter, more saturated fatty acids are positively, while lipid species containing longer, less saturated fatty acids are inversely, associated with MASLD. Lastly, we hypothesized an inverse association between betaine and MASLD. The results of our study will elucidate the roles of lipid and choline metabolism in MASLD, revealing potential biomarkers and interventional targets to reduce disease progression – a crucial consideration for MASLD, which is physiologically reversible.

## Methods

### Study sample

The CARDIA Study is a prospective cohort that began in 1985–86, with recruitment of 5,115 self-identified Black and White race men and women, aged 18–30 years from four U.S. urban centers: Birmingham, AL; Chicago, IL; Minneapolis, MN; and Oakland, CA [28]. A majority of the surviving cohort have attended 9 follow-up examinations [29]. Study participants provided informed consent at each examination and the Institutional Review Board at each CARDIA site approved exam protocols.

We used data from years 15, 20, and 25 of follow-up, at which data were generated on choline metabolites, plasma lipidome, and liver attenuation, respectively. Of the surviving cohort, 72% (n = 3,672) attended the Year 15 exam, 69% (n = 3,549) attended the Year 20 exam, and 68% (n = 3,499) attended the Year 25 exam; 2,711 participants had data on Y15 choline metabolites and Y25 liver attenuation, and 1,223 participants had data on Y20 plasma lipidomics and Y25 liver attenuation.

MASLD was defined based on liver attenuation scores derived from chest/abdominal CT scans conducted at Year 25 of follow-up. We excluded participants who, at Year 25, reported being pregnant; use of steatosis-inducing medication (amiodarone, methotrexate, valproate, and tamoxifen); reported history of hepatitis, cirrhosis, HIV, or heavy alcohol use (>14 drink/week for women, > 21 drink/week for men). After exclusions, 2,468 participants had data on choline metabolites and MASLD (n = 587), 1,116 had data on lipidomics and MASLD (n = 250), and 1,039 had data on choline metabolites, lipidomics, and MASLD (n = 234) (S1 Fig in S1 File). Other exclusions were based on missing covariates in regression analysis, as noted in relevant tables and figures.

### Blood sample collection and metabolite measurements

Participants were instructed to fast for ≥12 hours, and to avoid smoking and physical activity for 2 hours prior to the exam. Blood was drawn by venipuncture, separated through centrifugation, aliquoted, flash-frozen, and stored at −70◦ C. Plasma choline, betaine, and trimethylamine N-oxide (TMAO) were quantified by The University of North Carolina at Chapel Hill Nutrition Obesity Research Center Human Phenotyping Core using stable isotope dilution-liquid chromatography-multiple-reaction monitoring mass spectrometry [30,31]. An Atlantic HILIC Silica 3 mm 4.6x50mm column (Waters Corp) was used for chromatography, along with a Waters ACQUITY UPLC system (Waters Corp). For the mass spectrometric analysis, a Waters TQ detector (Waters Corp) equipped with an electrospray ionization probe set to positive-ion mode was used. The internal standards, TMAO-d9, choline-d9, and betaine-d9 were monitored at ion transitions of 85 > 66, 113 > 45, and 127 > 68 mass-to-charge ratios, respectively. Calibration curves constructed from the peak area ratios of the analyte to its internal standard allowed for quantification. The assay has a wide dynamic range (choline: 0.122–250 mM; betaine: 0.488–1000 mM; TMAO: 0.061–62.5 mM), high intra- and inter-day precision (CV < 6%), and high accuracy (<15% error).

Plasma lipids were quantified by Metabolon using infusion mass spectrometry. Lipids were extracted and concentrated using internal standards and nitrogen reconstituted in dichloromethane:methanol (1:1) with 10mM ammonium acetate. A Shimadzu LC with nano PEEK tubing and the Sciex SelexION-5500 QTRAP were used for infusion-MS in both positive and negative mode electrospray. Intensity ratios of target compounds and their assigned internal standards were used to quantify individual molecular lipid species. The targeted lipid panel quantified 14 lipid classes [triacyclglycerols (TAG), diacylglycerols (DAG), monoacylglycerols (MAG), ceramides (CER), hexosylceramides (HCER), lactosylceramides (LCER), dihydroceramides (DCER), phosphatidylcholines (PC), phosphatidylethanolamines (PE), phosphatidylinositols (PI), lysophosphatidylcholines (LPC), lysophosphatidylethanolamines (LPE), sphingomyelins (SM), and cholesteryl esters (CE)] and 756 molecular lipid species providing insight into FA composition within lipid class [e.g., PC(18:0/22:5)] [32]. FA composition, or the total carbons and double bonds present in a lipid, influences physiologic function of the lipid. Therefore, we derived a set of 300 lipid species as the sum of molecular lipid species that contained the same head group and had the same total number of carbons and double bonds [i.e., TAG(52:1)]. This transformation applied to lipid classes that contain >1 FA (DAG, PC, PE, PI, and TAG), and is consistent with standard lipidomic nomenclature and classifications proposed by the LIPID MAPS consortium [33].

### MASLD assessment

A non-contrast multidetector computed-tomography (CT) scan from either General Electric (GE 750HD 64 and GE LightSpeed VCT 64 Birmingham and Oakland Centers, respectively; GE Healthcare, Waukesha, WI) or Siemens (Sensation 64, Chicago and Minneapolis Centers; Siemens Medical Solutions, Erlangen, Germany) was used for abdominal CT. A core reading center (Wake Forest University Health Sciences, Winston‐Salem, NC) performed quality control and image analysis. The average of 9 measurements was used to calculate a mean liver attenuation score. MASLD was defined as a mean liver attenuation score <51 Hounsfield units. In a random sample of 156 participants, the interclass correlation coefficient for liver attenuation between different readers was 0.975, indicating high reproducibility of CT‐measured liver attenuation [34].

### Covariate measures

CARDIA utilized standard interviewer-administered questionnaires to assess sociodemographic characteristics (age, race, gender, education), pregnancy status (women only), smoking status, and medication use (including birth control, lipid-lowering, anti-hypertensive, and diabetes-related). Physical activity was assessed using a validated interviewer-administered CARDIA Physical Activity History questionnaire that includes engagement in 13 activities over the past-year. A total physical activity score was calculated by multiplying the frequency and intensity of each activity and summing over all activities [35].

CARDIA participants completed an interviewer-administered diet history at years 0, 7, and 20, to capture usual intake over the previous month [36]. The diet history captured previous months usual intake, coded into 166 food groups by the University of Minnesota’s Nutrition Coordinating Center, which were combined into 46 food groups. Food groups associated with at least one choline metabolite, or dietary precursors for choline metabolites, were included in analysis (eggs, processed red meat, lean red meat, regular red meat, chicken, fish, refined grains, whole grains, seeds and nuts, green vegetables, yellow vegetables, other vegetables, fruit, and fried foods). Additionally, we included total caloric intake and the previously described CARDIA *a priori* diet quality score (APDQS) [37].

Trained field center staff conducted clinical measures using standardized and validated protocols. BMI was calculated from in-clinic weight and height measures using a calibrated scale. Blood pressure was measured using a random zero sphygmomanometer three times and the mean of the second and third measures was used in analysis. Hypertension was defined by use of hypertension medications, or by a systolic pressure ≥ 140 mmHg or a diastolic pressure of ≥ 90 mmHg. For clinical lipid measures the Northwest Lipid Research Laboratory at the University of Washington used enzymatic procedures to quantify total cholesterol and triglycerides from fasted blood samples. The estimated glomerular filtration rate [eGFR (millimeter per minute per 1.32m^2^)] was calculated from serum creatinine, age, race, and sex using the 2009 Chronic Kidney Disease Epidemiology Collaboration equation [38].

### Statistical analysis

Molecular lipid species missing <25% were imputed with values generated from a random uniform distribution between the lowest and 1/10^th^ of the lowest observed concentrations for that specific lipid species. All lipid variables were log2 transformed and z-scaled. All choline metabolites were standardized to mean = 0, SD = 1. TMAO was natural log transformed for continuous analysis. For individual regression models we adjusted for covariates from the same exam as lipidomics (Year 20) or choline measures (Year 15). The basic model included field center (Birmingham, AL; Chicago, IL; Minneapolis, MN; Oakland, CA), age (continuous), sex (male/female), self-reported race (Black/White), and highest education attained (continuous years). The lifestyle model additionally adjusted for physical activity (continuous), smoking (current yes/no), and diet (food groups, APDQS, total energy). The clinical, or fully-adjusted, model additionally adjusted for eGFR (continuous), BMI (continuous), hypertension (yes/no), and lipid-lowering medication use (yes/no). P-values were adjusted for multiple comparisons using the false discovery rate (significance threshold q < 0.05).

Individual regression models, described above, tested associations between the 300 lipid species and MASLD. For TAG species, we used an additional model that adjusted for total TAGs to test the relationship between TAG FA composition and MASLD. This modeling method has previously shown that, when normalized to the total TAG content, TAGs containing longer, more unsaturated FAs are inversely associated with T2D [39]. We limited individual regression analysis to the 300 lipid species and reserved the 756 molecular lipid species for supervised analysis to increase statistical power in detecting significant associations and to increase interpretability of results with regard to total FA composition. In supervised models, statistical power is less concerning, allowing analysis of the 756 molecular lipid species which provides full FA identification [PC(16:0/16:1) vs. PC(32:1), for example]. Both lipid species and molecular lipid species are comparable to the lipidomic literature.

For supervised analysis of the molecular lipid species and MASLD we used logistic regression with LASSO penalty and a 0.7/0.3 training/testing split. Given a much larger number of non-cases in our sample, we implemented an oversampling approach commonly used in cases of imbalanced data to ensure that the variable selection process is more evenly driven by cases and non-cases [40]. Specifically, we implemented oversampling with the -ROSE- R package, to produce a case-balanced 0.7 training split. This approach uses a smoothed bootstrap approach to generate synthetic cases from conditional densities of cases and non-cases (n generated cases = 198) [41]. Given oversampling strategies are used to develop a better model within the training dataset, as recommended, oversampling was not applied to the 0.3 testing split (n cases/non-cases: training = 382/399, testing = 66/269). We derived a weighted MASLD-LRS using the absolute value of the beta coefficients from the penalized (LASSO) logistic regression. This MASLD-LRS is useful for 1) reducing molecular lipid species data, 2) accounting for high pairwise correlations, 3) simultaneously considering the lipidome which may be more relevant physiologically, and 4) associating the MASLD-LRS with choline metabolites and participant characteristics. We generated a table of descriptive statistics to characterize participants across tertiles of the MASLD-LRS.

We used individual regression models, as previously described, to test associations between choline metabolites and MASLD. Here, we included a fourth model that adjusted for the other two choline metabolites, in addition to model 3 covariates. Choline metabolites were modeled as continuous variables and secondly, as quartiles, to address potential non-linearity. Continuous modeling approaches assume that the effect of the metabolite on the outcome is consistent across the full distribution of the metabolite, whereas quartile modeling allows for the effect of the metabolite on the outcome to differ between each quartile. We note that these models, along with those testing associations between choline metabolites and the MASLD-LRS, may be viewed as an informal assessment of potential mediation of choline metabolite-MASLD associations by the plasma lipidome. Statistical analyses were conducted using R (version 4.2.3).

## Results

### Participant characteristics

MASLD was higher in men and individuals with adverse cardiometabolic risk profiles, with respect to hypertension, BMI, and lipid concentrations (Table 1). In the choline sample, 90% of MASLD cases, and in the lipidomic sample, 97% of MASLD cases, reported ≥ 1 or the cardiometabolic criteria (S1 Table in S1 File).

**Table 1:** Overall and metabolic associated steatotic liver disease-stratified participant characteristics^1^ among samples with data on choline metabolites (Y15) and metabolic associated steatotic liver disease (Y25), and lipidomics (Y20) and metabolic associated steatotic liver disease (Y25).

	Choline sample				Lipidomic sample			
	Overall	No MASLD	MASLD	p-value	Overall	No MASLD	MASLD	p-value
N	2468	1881	587		1116	866	250	
Choline (uM), Y15	8.53 (2.19)	8.37 (2.10)	9.05 (2.38)	<0.001	8.55 (2.19)	8.45 (2.17)	8.90 (2.22)	0.0025
Betaine (uM), Y15	37.61 (13.08)	37.83 (13.28)	36.89 (12.37)	0.077	37.30 (13.00)	37.58 (13.44)	36.36 (11.30)	0.18
ln-TMAO (uM), Y15	1.34 (1.10, 1.72)	1.34 (1.06, 1.69)	1.39 (1.13, 1.79)	<0.001	1.34 (1.06, 1.70)	1.31 (1.06, 1.67)	1.44 (1.16, 1.81)	0.0018
Exam Age (Y0)	25.16 (3.61)	25.08 (3.61)	25.41 (3.59)	0.045	25.16 (3.61)	25.08 (3.60)	25.44 (3.65)	0.12
Race, % white	54	53	56	0.26	57	56	58	0.59
Sex, % female	57	61	44	<0.001	58	64	39	<0.001
Max education attained (yrs.)	16.00 (14.00, 18.00)	16.00 (14.00, 18.00)	16.00 (14.00, 17.00)	0.022	16.00 (14.00, 18.00)	16.00 (14.00, 18.00)	16.00 (14.00, 18.00)	0.41
Current smoking, % yes	19	19	21	0.29	15	14	17	0.31
Physical activity score	279 (141, 491)	285 (142, 498)	270 (138, 467)	0.27	276 (130, 489)	281 (125, 501)	251 (146, 480)	0.63
BMI (kg/m2)	27.49 (24.10, 32.26)	26.33 (23.38, 30.33)	31.99 (28.06, 36.43)	<0.001	28.14 (24.59, 32.68)	26.84 (23.88, 30.94)	32.55 (29.03, 36.74)	<0.001
High blood pressure, % yes	16	13	23	<0.001	20	16	33	<0.001
Lipid-lowering medication, % yes	2	1.9	2.6	0.38	8.7	7	15	<0.001
Total cholesterol (mg/dL)	182.00 (161.00, 205.00)	180.00 (160.00, 203.00)	189.50 (166.00, 212.00)	<0.001	185.09 (34.16)	185.06 (33.87)	185.19 (35.18)	0.86
Triglycerides (mg/dL)	82.00 (59.00, 121.00)	76.00 (55.00, 108.00)	114.00 (79.00, 163.75)	<0.001	84.00 (62.00, 122.00)	77.00 (58.00, 107.00)	119.00 (86.00, 172.00)	<0.001
eGFR	103.48 (16.50)	103.86 (16.17)	102.27 (17.47)	0.024	95.99 (82.75, 104.34)	96.12 (82.75, 104.87)	95.54 (84.92, 103.81)	0.75
Mean liver attenuation (HU)	57.87 (51.51, 62.39)	60.01 (56.39, 63.98)	42.65 (32.99, 47.74)	<0.001	58.42 (51.97, 63.07)	60.24 (56.78, 64.67)	42.05 (32.92, 47.23)	<0.001

*BMI* body mass index, *eGFR* estimated glomerular filtration rate, *MASLD* metabolic associated steatotic liver disease, *TMAO* trimethylamine N-oxide.

### Lipids and MASLD

**Fig 1:**
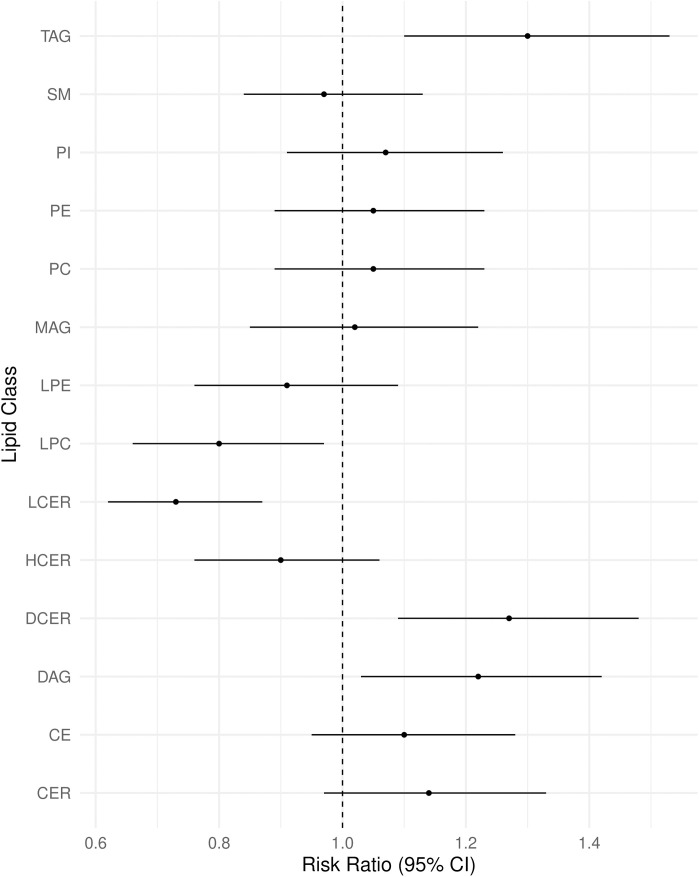
Multivariable-adjusted associations between lipid classes and metabolic associated steatotic liver disease. Multivariable-adjusted risk ratios (95% CI) from a Poisson regression model adjusted for race (Black/White), examination center (Birmingham, AL; Chicago, IL; Minneapolis, MN; Oakland, CA), age (continuous), attained education (continuous), sex (male/female), smoking (current/not current), physical activity score (continuous), dietary intake as food groups (continuous), caloric intake (continuous), a priori diet quality score (continuous), eGFR (continuous), BMI (continuous), hypertension (yes/no), and lipid-lowering cholesterol medication use (yes/no). MASLD cases/non-cases = 219/764; Observations removed due to missing covariate data = 133. *CE* cholesteryl ester, *CER* ceramide, *DAG* diacylglycerol, *DCER* dihydroceramide, *HCER* hexosylceramide, *LCER* Lactosylceramide, *LPE* lysophosphatidylcholine, *MAG* monoacylglycerol, *PC* phosphatidylcholine, *PE* phosphatidylethanolamine, *PI* phosphatidylinositol, *SM* sphingomyelin, *TAG* triacylglycerol.

**Fig 2:**
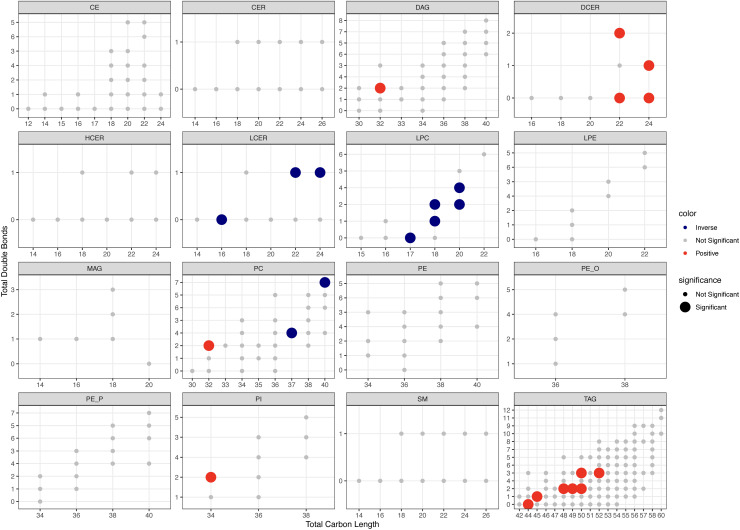
Multivariable-adjusted associations between lipid species and metabolic associated steatotic liver disease. Multivariable-adjusted risk ratios (95% CI) from a Poisson regression model adjusted for race (Black/White), examination center (Birmingham, AL; Chicago, IL; Minneapolis, MN; Oakland, CA), age (continuous), attained education (continuous), sex (male/female), smoking (current/not current), physical activity score (continuous), dietary intake as food groups (continuous), caloric intake (continuous), a priori diet quality score (continuous), eGFR (continuous), BMI (continuous), hypertension (yes/no), and lipid-lowering cholesterol medication use (yes/no). n cases/non-cases = 219/764; observations removed due to missing covariate data = 133. *CE* cholesteryl ester, *CER* ceramide, *DAG* diacylglycerol, *DCER* dihydroceramide, *HCER* hexosylceramide, *LCER* Lactosylceramide, *LPE* lysophosphatidylcholine, *MAG* monoacylglycerol, *PC* phosphatidylcholine, *PE* phosphatidylethanolamine, *PE_O* phosphatidylethanolamine ether, *PE_P* phosphatidylethanolamine plasmalogen, *PI* phosphatidylinositol, *SM* sphingomyelin, *TAG* triacylglycerol.

Six of 14 lipid classes were associated with MASLD in multivariable-adjusted regression. Total TAGs, DAGs, CERs, and DCERs were positively, and LPCs and LCERs inversely, associated with MASLD (Fig 1), of which associations for TAGs, DAGs, DCERs, and LCERs had q-values<0.05 (S2 Table in S1 File). In multivariable-adjusted regression of 300 lipid species with more than one double bond, 24 species were positively, and 10 species were inversely, associated with MASLD. DCERs(22:0, 22:2, 24:0, and 24:1), DAG(32:2), PC(32:2), and PI(34:2) were positively associated with MASLD. LCER(16:0, 22:1, and 24:1), LPC(17:0, 18:1, 18:2, 20:2, and 20:4), and PC(37:4 and 40:7) were inversely associated with MASLD (Fig 2 and S3 Table in S1 File). 7 TAGs containing shorter and more saturated FAs (total carbon length <52 and total double bonds <4) were positively associated with MASLD while TAGS containing longer, more unsaturated FAs were not associated with MASLD (Fig 2). In a model adjusting for total TAGs, TAGs containing longer, more unsaturated FAs, with total carbon length >52 and total double bonds >2, were inversely associated with MASLD (S3 Fig in S1 File).

### MASLD-lipid risk score

**Fig 3:**
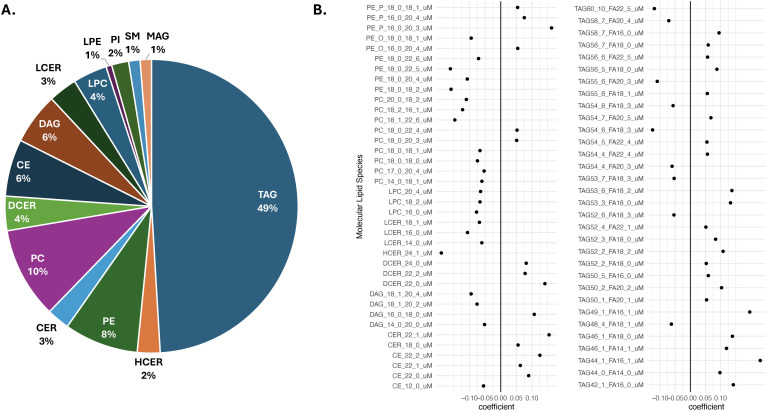
Composition of the weighted metabolic associated steatotic liver disease-lipidomic risk score derived from a penalized (least absolute shrinkage and selection operator) regression of 756 molecular lipid species and metabolic associated steatotic liver disease at the A. class-level, and B. the species level. Metabolic associated steatotic liver disease-lipidomic risk score (MASLD-LRS) = lasso coefficient*concentration/ total # of significant lipids. Penalized (least absolute shrinkage and selection operator) regression model trained on a 0.7 case-oversampled subset and tested on the remaining 0.3 (71% sensitivity, 70% specificity, 0.70 AUC). Molecular lipid species displayed are those with a coefficient > |0.5| in the penalized (least absolute shrinkage and selection operator) regression. *CE* cholesteryl ester, *CER* ceramide, *DAG* diacylglycerol, *DCER* dihydroceramide, *HCER* hexosylceramide, *LCER* Lactosylceramide, *LPE* lysophosphatidylcholine, *MAG* monoacylglycerol, *PC* phosphatidylcholine, *PE* phosphatidylethanolamine, *PE_O* phosphatidylethanolamine ether, *PE_P* phosphatidylethanolamine plasmalogen, *PI* phosphatidylinositol, *SM* sphingomyelin, *TAG* triacylglycerol.

An unadjusted penalized (LASSO) logistic regression model distinguished individuals with and without MASLD with 71% sensitivity,70% specificity, and 0.70 AUC in the testing set. We used this model to generate a MASLD lipidomic risk score (MASLD-LRS) from the molecular lipid species. The LASSO penalty selected 159 molecular lipid species that distinguished MASLD cases from non-cases (S4 Table in S1 File). Using these beta-coefficients we developed a weighted MASLD-LRS comprised primarily of TAGs, PCs, and PEs (49%, 10%, and 8%, respectively) (Fig 3). In total, 70 molecular lipid species of the MASLD-LRS had model coefficients > |0.05| and included 32 TAGs, 9 PCs, 9 PEs, 5 CEs, 4 DAGs, 3 DCERs, 3 LCERs, 3 LPCs, 1 CER, and 1 LPE (Fig 3). Lastly, we generated a table of descriptive statistics stratified by tertiles of the MASLD-LRS. Participants who reported male sex, had higher BMIs, hypertension, use lipid-lowering medications, and lower APDQSs were more likely to have a higher MASLD-LRS (S5 Table in S1 File).

### Choline metabolites and MASLD

Next, we tested associations between choline metabolites and MASLD since choline is a precursor of phospholipids, holding a significant role in lipid transport and metabolism [42]. In the fully-adjusted model (including basic sociodemographics, lifestyle, and clinical variables), betaine was inversely associated with MASLD, with a 0.83 (0.74, 0.91) risk ratio for a standard deviation higher betaine concentration. These findings were robust to model specifications and form of betaine as quartiles. Results for choline and TMAO were sensitive to model specifications and variable format: neither metabolite was associated with MASLD when modeled continuous; but, for both metabolites, quartile 4 (highest level), compared to quartile 1 (lowest level), was significantly positively associated with MASLD (Table 2).

**Table 2:** Multivariable adjusted associations^1^ [risk ratio (95% CI)] between choline metabolites and MASLD.

		Metabolite Quartiles				Continuous metabolites (per 1-SD increase)
	Model^1,2,3,4^	Q1 (lowest; REF)	Q2	Q3	Q4 (highest)	
Choline	1	1	1.22 (0.94, 1.58)	1.38 (1.08, 1.78)	1.60 (1.26, 2.05)	1.17 (1.09, 1.26)
	2	1	1.17 (0.90, 1.52)	1.34 (1.04, 1.73)	1.55 (1.21, 1.99)	1.17 (1.08, 1.25)
	3	1	1.03 (0.79, 1.34	1.08 (0.84, 1.41)	1.12 (0.87, 1.45)	1.02 (0.94, 1.10)
	4	1	1.09 (0.83, 1.42)	1.23 (0.94, 1.60)	1.37 (1.05, 1.81)	1.09 (1.00, 1.19)
Betaine	Model ^1,2,3,4^					
	1	1	1.03 (0.83, 1.29)	0.66 (0.51, 0.84)	0.65 (0.51, 0.84)	0.83 (0.75, 0.91)
	2	1	1.03 (0.82, 1.29)	0.66 (0.51, 0.85)	0.7 (0.54, 0.90)	0.85 (0.77, 0.93)
	3	1	1.04 (0.83, 1.31)	0.71 (0.55, 0.92)	0.76 (0.59, 0.99)	0.87 (0.79, 0.95)
	4	1	1.01 (0.81, 1.28)	0.67 (0.52, 0.87)	0.69 (0.52, 0.91)	0.83 (0.74, 0.91)
TMAO^3^	Model ^1,2,3,4^					
	1	1	1.34 (1.06, 1.72)	1.2 (0.93, 1.54)	1.37 (1.07, 1.74)	1.10 (1.02, 1.19)
	2	1	1.38 (1.08, 1.77)	1.20 (0.93, 1.55)	1.35 (1.06, 1.73)	1.09 (1.01, 1.18)
	3	1	1.40 (1.09, 1.81)	1.17 (0.90, 1.51)	1.34 (1.04, 1.72)	1.08 (0.99, 1.17)
	4	1	1.39 (1.09, 1.80)	1.16 (0.90, 1.51)	1.32 (1.03, 1.70)	1.07 (0.99, 1.15)

^1^Poisson regression models adjusted for 1 = race (Black/White), examination center (Birmingham, AL; Chicago, IL; Oakland, CA; Minneapolis, MN), age (continuous), attained education (continuous), sex (male/female); 2 = additionally adjusted for smoking (current/not current), physical activity score (continuous), dietary intake as food groups (continuous), caloric intake (continuous), a priori diet quality score (continuous); 3 = additionally adjusted for eGFR (continuous), BMI (continuous), hypertension (yes/no), lipid-lowering cholesterol medication use (yes/no); 4 = additionally adjusted for other two choline metabolites. ^2^n cases/non-cases: M1 = 587/1881, M2 = 573/1843, M3 = 566/1821, M4 = 566/1821. ^3^Trimethylamine n-oxide (TMAO) natural log transformed in analysis of continuous metabolites.

### Lipidomics and choline metabolites

Correlation coefficients were: choline and betaine = 0.42, choline and ln-TMAO = 0.08, and betaine and ln-TMAO = 0.04. Many lipid classes were correlated, either positively or inversely (S5 Fig in S1 File). In the fully-adjusted regression model, choline was positively associated with TAGs, PEs, PCs, LPEs, LPCs, DCERs, DAGs, and CERs; betaine was positively associated with MAGs, LPCs, LCERs, and HCERs, and inversely associated with TAGs, PEs, PCs, and DAGs; and ln-TMAO was not associated with any lipid class (S6 Fig in S1 File). Choline was positively associated, betaine was inversely associated, and TMAO was not associated with the derived MASLD-LRS (S7 Fig in S1 File). Significant betaine associations with both MASLD and the MASLD-LRS, suggesting betaine is associated with a lipidomic profile which explains variation in those with and without MASLD, are supportive of mediation of the betaine-MASLD relation by the lipidome.

## Discussion

Continuing advancements in lipidomic technologies have yielded high-dimensional lipidomic data that can provide insights into the metabolic alterations associated with MASLD. Using population-based lipidomic data, we identified class- and species-level lipidome features and choline metabolites that were associated with MASLD in a U.S. cohort of early-middle-aged adults. As hypothesized, we replicated positive associations between total TAGs, DAGs, and CERs with MASLD, but not total PCs. We found diverging associations for lipid species within the PC class, highlighting unique value of species-level lipidomic data. These species-level results were consistent with hypothesized positive MASLD associations for PCs containing shorter, more saturated fatty acids and inverse MASLD associations for PCs containing longer, less saturated fatty acids. This finding is mechanistically supported as longer, polyunsaturated PCs may be specifically beneficial in reducing reduce hepatic TAG accumulation through lipoprotein metabolism [24]. We further identified an inverse association between betaine and MASLD, which aligns with published mechanistic evidence [11–13]. Our finding that the MASLD-LRS was positively associated with choline, and inversely associated with betaine, provides quantitative support for connections between choline metabolism and the MASLD lipidome. Together these results support further investigation into the role of choline metabolites in lipid metabolism and the development of MASLD, as well as the potential for these metabolites to serve as biomarkers of MASLD. To our knowledge, this investigation contributes the first population-based results for lipidomics and choline metabolites in MASLD.

Choline metabolism is known to relate to liver health, as humans and animals fed choline-deficient diets develop liver dysfunction [43,44]. Choline is an essential nutrient that is metabolized to either PC or betaine in the liver. As the most abundant phospholipid, PC concentrations are generally stable unless choline intake is very low [43,45]; in contrast, betaine concentrations are more sensitive to changes in choline intake [45]. Increased plasma betaine, a proposed marker of high choline intake, has demonstrated beneficial roles in lipogenic gene suppression, insulin signaling, and modulation of inflammation and oxidative stress [13,46–48]. Our association between betaine, the lipid classes, and the MASLD-LRS is supported by these mechanisms, as betaine may play a significant role in improving lipid profiles. The inverse association between betaine and MASLD has been documented in other published results that vary in study design and sample characteristics [14,15]. Unlike betaine, total PC concentrations are not as affected by choline intake, nor were they associated with MASLD in our study. However, the effect of choline intake on PC species, particularly those that were associated with MASLD, is understudied. While our study lacks data on choline intake to support the potential benefits of high choline intake on lipid species and MASLD, a previous pilot study found that a choline and betaine nutrient cocktail reduced measures of liver steatosis and fibrosis [49]. Together, our results and existing evidence suggests that betaine plays a beneficial in lipid metabolism to reduce MASLD and supports further investigation of dietary choline’s impact on PC species, betaine, and MASLD.

Unlike other published lipidomic studies, we tested associations at both the class- and species-level of the plasma lipidome, offering comprehensive insight into the MASLD lipidome, and identifying differences between class- and species-level results [16,21,50–52]. We observed three scenarios where class- and species-level results differed, highlighting the value of comparing class- and species-level analyses. For PCs, PIs, and CERs, we noted three scenarios where class- and species-level results differed. First, species-level analysis may reveal associations not observed in class-level analysis. For example, we identified a significant association for PI species PI(34:2), but total PIs were not associated. Second, class-level analysis may reveal an association, while species-level analysis may not. We observed this for CERs, which were in positively associated in class-level, but not in species-level analysis. Third species within a class may be associated in opposite directions and average out to a null association in class-level analysis. For example, we observed diverging effects of lipid species within PCs, nullifying the class-level association for PCs and MASLD. Similarly, we noted that the positive association for TAGs at the class-level was driven by TAG species comprised of shorter, more saturated FAs. Other lipid classes did not indicate significant differences by FA composition and further study is needed to elucidate whether these FA-specific effects are limited to PCs and TAGs, or if other lipid classes are implicated.

Our investigation of the plasma lipidome, choline metabolites, and MASLD in the CARDIA study has many strengths that add to the increasing evidence base of MASLD. A primary strength is that we used population-based data from a large U.S. based cohort of early-middle aged adults; increasing the generalizability of findings to younger and healthier individuals, when cardiometabolic risk is increasing and interventions may be most effective. Other studies of MASLD have been limited to analysis of clinic-based or small samples, narrow lipidome coverage, and lack data on both choline metabolites and lipidomics [14,15,53,54]. In our sample, fewer cases met MASLDs cardiometabolic criteria, reflecting the nature of CARDIAs population-based sampling and younger age, in contrast to existing clinic-based studies. In addition, the CARDIA study is a unique data source that includes clinical assessment of cardiometabolic risk factors, providing extensive data on potential confounders to include in multivariable-adjusted models. Another strength of our study is that we derived a population-based MASLD-LRS from molecular species data, which can be utilized to strengthen subsequent population-based lipidomic studies and inform the prognostic value of molecular species data. A limitation of our study is that metabolite measures were from a single fasting blood sample, though lipidomic measures from human plasma have shown high reproducibility and stability [55,56]. Other studies using targeted lipidomic platforms often include fewer molecular lipid species than the platform we used which fully quantified 756 molecular lipid species across 14 lipid classes. However, this targeted platform is unable to capture the full complexity of lipid metabolism as many other lipid related metabolites exist that are implicated in MASLD, particularly free fatty acids and oxidative lipid products. In addition, the CARDIA nutrient database does not currently include dietary choline intake, which is needed to further support claims that high choline intake may benefit MASLD through increasing plasma betaine. While choline is rapidly converted to either betaine or PC in the liver and plasma concentrations are subject to dietary intake, choline metabolite concentrations from fasted plasma samples display high reliability [57–59]. On the other hand, identifying non-invasive approaches to defining MASLD is an active area of research and CT measures, like those used in our study, may lack sensitivity [60,61]. Our exposure and outcome measures were captured at single time points, with assessment of plasma choline metabolites and lipidome at exams 5- and 10-years prior to MASLD, respectively. This design may contribute extraneous variability, as compared to concurrent measurements, which may attenuate results. Although metabolite exposures were assessed before MASLD, there remains a risk of reverse causality, to the extent that fatty liver was present 5–10 years before MASLD assessment. Longitudinal data with concomitantly assessed metabolite and fatty liver measurement, and follow-up assessment of fatty liver, are needed for rigorous replication of our findings. Despite these limitations in our design, our data contributes to efforts in identifying population-based biomarkers of MASLD, which has been a broadly recognized need, since the current standard for MASLD diagnosis requires imaging or liver biopsy.

In conclusion, we identified lipidomic and choline metabolite signatures of MASLD in a population-based study of U.S. adults, demonstrated the importance of class- and species-level lipidomic data, and documented links between choline and lipid metabolism in MASLD. Class- and species-level results support diverging roles of PC species based on FA composition as PC(37:4) and PC(40:7) were inversely, and PC(32:2) was positively associated with MASLD. Given the mechanisms of PCs in hepatic lipid metabolism, these species have potential as biomarkers of MASLD, though the specificity of these species to MASLD, and not other cardiometabolic diseases may be limiting [62,63]. A LRS that simultaneously considers the lipidome features implicated in MASLD, like our derived MASLD-LRS, may have greater specificity for MASLD than individual lipid species. However, large studies with validation cohorts are required to test this. Other factors that could influence whether these species, and our MASLD-LRS, can serve as biomarkers of MASLD include dietary intake and genetics in choline and lipid-metabolism. Future lipidomic studies of MASLD should consider these variables for precision nutrition and lifestyle intervention efforts to reduce MASLD. Collectively, our conclusions further efforts in biomarker identification, elucidate the links between choline and lipid metabolites in MASLD, and support further longitudinal population-based studies of the lipidome and MASLD with consideration of factors that may favorably modify the plasma lipidome.

## Supporting information

S1 File Supporting Information. (DOCX)
