# Supplementary material for: Plasma lipidomics, choline metabolites, and metabolic-associated steatotic liver disease (MASLD): A Coronary Artery Risk Development in Young Adults (CARDIA) study
Source: PLoS One. 2026 Mar 13;21(3):e0341462. doi: 10.1371/journal.pone.0341462 (PMC12987446; doi:10.1371/journal.pone.0341462)
Supplement: S1 File — (DOCX) [file pone.0341462.s001.docx]

Supplementary Materials

Supplemental figure 1. Study design flowchart


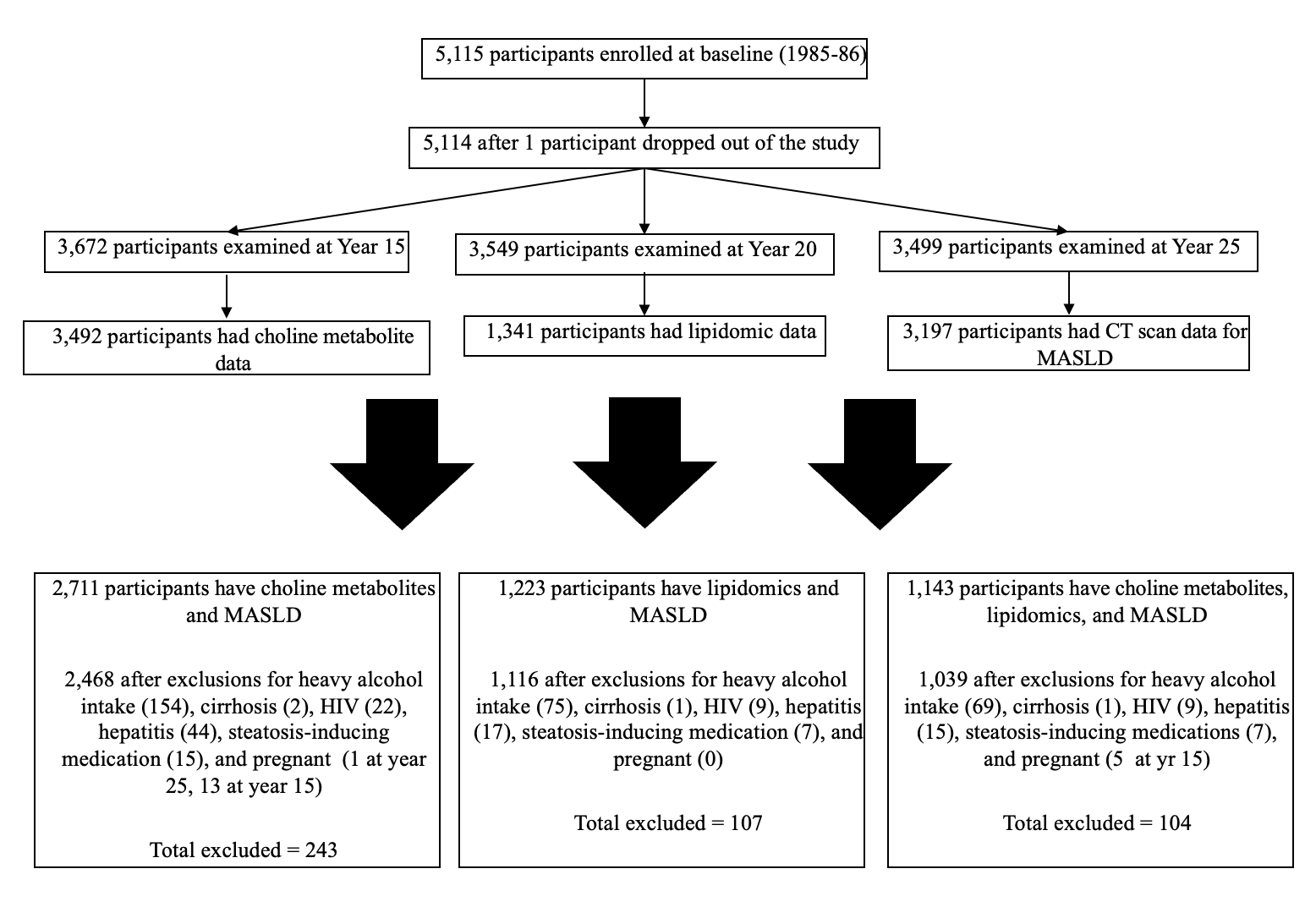


Supplemental table 1. MASLD cardiometabolic criteria^1^

|  | **Choline sample** | | | | **Lipidomic sample** | | | |
| --- | --- | --- | --- | --- | --- | --- | --- | --- |
|  | **Overall** | **No MASLD** | **MASLD** | **p-value** | **Overall** | **No MASLD** | **MASLD** | **p-value** |
| BMI >= 25kg/m^2^, % | 70 | 65 | 87 | <0.001 | 73 | 67 | 96 | <0.001 |
| Insulin resistance^2^, % | 36 | 30 | 56 | <0.001 | 37 | 30 | 59 | <0.001 |
| Hypertension^3^, % | 45 | 40 | 60 | <0.001 | 43 | 37 | 62 | <0.001 |
| High triglycerides^4^, % | 29 | 23 | 48 | <0.001 | 28 | 21 | 54 | <0.001 |
| Dyslipidemia^5^, % | 32 | 27 | 48 | <0.001 | 33 | 27 | 54 | <0.001 |
| Prevalence of >= 1 cardiometabolic criteria, % | 81 | 78 | 90 | <0.001 | 84 | 80 | 97 | <0.001 |

^1^MASLD cardiometabolic criteria defined by Hagstrom, et. al.

^2^Fasting glucose >= 5.6 mM, or HbA1c >= 39 mM, or known diabetes, or antidiabetic medication use.

^3^Blood pressure >=130/85 mmHg, or antihypertensive medication use.

^4^Plasma triglycerides >=1.7mM, or lipid-lowering medication use.

^5^HDL-c <= 1.0mM for men and <= 1.3mM for women, or lipid-lowering medication use.

Supplemental table 2. Multivariable-adjusted risk ratios^1^ (95% CI) for lipid classes and MASLD.

|  | Model 1 | | Model 2 | | Model 3 | |
| --- | --- | --- | --- | --- | --- | --- |
| Lipid Class | RR (95% CI) | p-value^2^ | RR (95% CI) | p-value | RR (95% CI) | p-value |
| DCER | 1.47 (1.3, 1.67) | <0.001 | 1.41 (1.23, 1.61) | <0.001 | 1.32 (1.16, 1.51) | <0.001 |
| TAG | 1.57 (1.4, 1.77) | <0.001 | 1.49 (1.31, 1.69) | <0.001 | 1.33 (1.15, 1.53) | <0.001 |
| DAG | 1.5 (1.33, 1.68) | <0.001 | 1.43 (1.26, 1.62) | <0.001 | 1.28 (1.12, 1.47) | 0.0037 |
| LCER | 0.66 (0.59, 0.75) | <0.001 | 0.7 (0.61, 0.8) | <0.001 | 0.78 (0.68, 0.9) | 0.0064 |
| LPC | 0.63 (0.55, 0.74) | <0.001 | 0.68 (0.58, 0.79) | <0.001 | 0.8 (0.68, 0.94) | 0.0594 |
| CER | 1.2 (1.06, 1.36) | 0.0127 | 1.16 (1.01, 1.32) | 0.1857 | 1.17 (1.02, 1.34) | 0.1436 |
| CE | 1.06 (0.94, 1.19) | 0.5083 | 1.06 (0.93, 1.2) | 0.7902 | 1.08 (0.95, 1.22) | 0.7351 |
| HCER | 0.77 (0.68, 0.87) | <0.001 | 0.79 (0.7, 0.9) | 0.0065 | 0.91 (0.8, 1.05) | 0.7351 |
| LPE | 0.82 (0.71, 0.95) | 0.0245 | 0.84 (0.72, 0.98) | 0.1594 | 0.9 (0.77, 1.05) | 0.7351 |
| PI | 1.12 (0.99, 1.27) | 0.131 | 1.09 (0.95, 1.25) | 0.7296 | 1.09 (0.95, 1.25) | 0.7351 |
| PC | 1.05 (0.92, 1.19) | 0.6152 | 1.03 (0.9, 1.18) | 0.8503 | 1.07 (0.93, 1.23) | 0.9066 |
| MAG | 1 (0.88, 1.15) | 0.9559 | 1.03 (0.89, 1.19) | 0.861 | 1.04 (0.89, 1.21) | 0.9989 |
| PE | 1.06 (0.94, 1.2) | 0.4782 | 1.05 (0.91, 1.2) | 0.826 | 1.04 (0.91, 1.2) | 0.9989 |
| SM | 0.92 (0.81, 1.04) | 0.2955 | 0.93 (0.82, 1.06) | 0.7501 | 0.98 (0.86, 1.11) | 0.9989 |

^1^Individual Poisson log-risk regression models adjusted for: M1 = race, CARDIA center, age, education, and sex; M2 = M1 + smoking, physical activity, dietary intake (food groups, total energy, and APDQS); M3 = M2 + eGFR, BMI, hypertension, and lipid-lowering medication use.

^2^P-values adjusted for the false discovery rate using the Benjamin-Hochberg procedure.

Supplemental table 3. Multivariable-adjusted risk ratios^1^ (95% CI) for lipid species^2^ and MASLD.

| Lipid species | RR (95% CI) | p-value | Adjusted p-value^3^ |
| --- | --- | --- | --- |
| LCER 24 :1 | 0.7 (0.59, 0.83) | 3.17009E-05 | 0.000445195 |
| DCER 22 :0 | 1.25 (1.12, 1.4) | 5.30242E-05 | 0.000627918 |
| LPC 18 :1 | 0.67 (0.55, 0.81) | 6.97987E-05 | 0.00078269 |
| PI 34 :2 | 1.23 (1.09, 1.39) | 0.000630231 | 0.006281373 |
| DCER 22 :2 | 1.21 (1.08, 1.34) | 0.000768463 | 0.007642171 |
| LCER 16 :0 | 0.76 (0.65, 0.89) | 0.000779139 | 0.007731259 |
| LPC 20 :2 | 0.77 (0.65, 0.9) | 0.0008807 | 0.008729406 |
| LPC 17 :0 | 0.73 (0.6, 0.88) | 0.001272575 | 0.012544552 |
| DCER 24 :0 | 1.22 (1.08, 1.38) | 0.0013559 | 0.013264236 |
| LCER 22 :1 | 0.78 (0.67, 0.91) | 0.001560328 | 0.014860268 |
| LPC 18 :2 | 0.74 (0.61, 0.89) | 0.001632133 | 0.015253582 |
| LPC 20 :4 | 0.74 (0.61, 0.89) | 0.001651464 | 0.015338672 |
| PC 40 :7 | 0.76 (0.64, 0.9) | 0.001680815 | 0.015421152 |
| DCER 24 :1 | 1.22 (1.07, 1.37) | 0.001715483 | 0.015446973 |
| TAG 45 :1 | 1.15 (1.04, 1.25) | 0.002002153 | 0.016227478 |
| TAG 50 :2 | 1.16 (1.04, 1.27) | 0.003512451 | 0.02614728 |
| TAG 50 :3 | 1.17 (1.05, 1.3) | 0.003582597 | 0.026625409 |
| DAG 32 :2 | 1.17 (1.04, 1.29) | 0.004643949 | 0.034371331 |
| PC 32 :2 | 1.25 (1.07, 1.46) | 0.005023617 | 0.037089868 |
| TAG 44 :0 | 1.14 (1.03, 1.24) | 0.005457343 | 0.04025909 |
| TAG 49 :2 | 1.16 (1.04, 1.29) | 0.00569643 | 0.041988425 |
| TAG 52 :3 | 1.16 (1.04, 1.28) | 0.005905668 | 0.043495101 |
| TAG 48 :2 | 1.16 (1.04, 1.28) | 0.006027121 | 0.044353304 |
| PC 37 :4 | 0.81 (0.69, 0.94) | 0.006077046 | 0.04468416 |
| LCER 14 :0 | 0.76 (0.63, 0.93) | 0.007290162 | 0.053516685 |
| TAG 50 :1 | 1.13 (1.02, 1.23) | 0.00730736 | 0.053599218 |
| DCER 18 :0 | 1.17 (1.04, 1.31) | 0.007940895 | 0.058198745 |
| TAG 50 :4 | 1.16 (1.03, 1.3) | 0.009014172 | 0.066011024 |
| CER 22 :0 | 1.17 (1.03, 1.32) | 0.01018492 | 0.074463286 |
| TAG 48 :1 | 1.14 (1.02, 1.25) | 0.0102916 | 0.075182121 |
| TAG 48 :3 | 1.14 (1.02, 1.25) | 0.0104006 | 0.075916782 |
| HCER 24 :1 | 0.81 (0.68, 0.95) | 0.01045656 | 0.07626337 |
| TAG 49 :3 | 1.16 (1.03, 1.29) | 0.01109641 | 0.080864501 |
| TAG 51 :3 | 1.16 (1.03, 1.29) | 0.01112388 | 0.080999134 |
| TAG 52 :4 | 1.15 (1.03, 1.28) | 0.01140205 | 0.082957503 |
| TAG 49 :1 | 1.14 (1.02, 1.26) | 0.01210344 | 0.087918441 |
| CER 18 :0 | 1.17 (1.03, 1.32) | 0.01363031 | 0.098929667 |
| TAG 52 :2 | 1.13 (1.01, 1.24) | 0.01381132 | 0.100162642 |
| DAG 34 :2 | 1.13 (1.02, 1.25) | 0.01493196 | 0.108202638 |
| TAG 47 :2 | 1.13 (1.02, 1.25) | 0.01707743 | 0.123451302 |
| DAG 30 :0 | 1.13 (1.01, 1.24) | 0.01774202 | 0.128049849 |
| TAG 46 :0 | 1.12 (1.01, 1.23) | 0.01813964 | 0.130501038 |
| PE 38 :3 | 1.17 (1.02, 1.33) | 0.01852376 | 0.132733918 |
| DAG 36 :6 | 1.15 (1.02, 1.29) | 0.02013191 | 0.142330862 |
| LPE 18 :0 | 0.82 (0.68, 0.97) | 0.02166007 | 0.150999702 |
| TAG 51 :2 | 1.13 (1.01, 1.25) | 0.02243662 | 0.154616856 |
| TAG 55 :7 | 1.13 (1.01, 1.24) | 0.02249106 | 0.154775607 |
| TAG 50 :5 | 1.14 (1.01, 1.27) | 0.02252812 | 0.154775607 |
| LCER 18 :1 | 0.84 (0.72, 0.97) | 0.02254565 | 0.154775607 |
| TAG 45 :0 | 1.11 (1.01, 1.21) | 0.02330039 | 0.157081255 |
| TAG 47 :1 | 1.13 (1.01, 1.24) | 0.02338634 | 0.157542726 |
| TAG 53 :0 | 1.13 (1.01, 1.25) | 0.0245794 | 0.160393961 |
| LPC 18 :0 | 0.81 (0.67, 0.97) | 0.02522776 | 0.161178757 |
| DAG 34 :3 | 1.14 (1.01, 1.26) | 0.02579382 | 0.161178757 |
| TAG 46 :1 | 1.12 (1.01, 1.22) | 0.02600658 | 0.161178757 |
| TAG 52 :5 | 1.14 (1.01, 1.27) | 0.02643532 | 0.161250054 |
| TAG 51 :4 | 1.14 (1.01, 1.27) | 0.02811041 | 0.166882355 |
| TAG 53 :6 | 1.15 (1.01, 1.29) | 0.02999428 | 0.175886841 |
| CE 16 :1 | 1.15 (1.01, 1.3) | 0.03042654 | 0.177933007 |
| PC 38 :3 | 1.15 (1.01, 1.31) | 0.03071352 | 0.17937813 |
| TAG 46 :2 | 1.1 (1, 1.2) | 0.03120056 | 0.181633292 |
| TAG 52 :8 | 1.12 (1, 1.23) | 0.03126388 | 0.181884249 |
| DAG 32 :1 | 1.12 (1, 1.22) | 0.03163476 | 0.18356726 |
| CE 14 :1 | 1.15 (1.01, 1.31) | 0.03209251 | 0.185863961 |
| CE 20 :3 | 1.15 (1.01, 1.3) | 0.0323487 | 0.187227178 |
| TAG 57 :9 | 1.16 (1.01, 1.32) | 0.03261932 | 0.188672153 |
| LCER 24 :0 | 0.84 (0.71, 0.98) | 0.03362701 | 0.194126393 |
| PC 35 :1 | 0.85 (0.73, 0.99) | 0.03555551 | 0.204472599 |
| TAG 48 :4 | 1.1 (1, 1.2) | 0.03630649 | 0.208658011 |
| CE 14 :0 | 1.16 (1.01, 1.32) | 0.0367771 | 0.211093059 |
| DAG 32 :3 | 1.12 (1, 1.23) | 0.03849124 | 0.220650442 |
| TAG 55 :1 | 1.13 (1, 1.27) | 0.03865263 | 0.221434552 |
| DAG 33 :1 | 1.12 (1, 1.24) | 0.03978466 | 0.227774768 |
| TAG 46 :3 | 1.11 (1, 1.21) | 0.04059871 | 0.232287595 |
| TAG 53 :5 | 1.12 (1, 1.24) | 0.04072033 | 0.232835408 |
| CER 24 :0 | 1.15 (1, 1.3) | 0.04158436 | 0.237608683 |
| TAG 52 :1 | 1.1 (0.99, 1.19) | 0.04200779 | 0.239588166 |
| TAG 48 :0 | 1.11 (0.99, 1.21) | 0.04340137 | 0.247379591 |
| TAG 50 :0 | 1.1 (0.99, 1.2) | 0.04512694 | 0.257052174 |
| TAG 42 :0 | 1.1 (0.99, 1.21) | 0.04535336 | 0.258178503 |
| TAG 51 :1 | 1.11 (0.99, 1.21) | 0.04544897 | 0.258559239 |
| DAG 30 :1 | 1.13 (1, 1.27) | 0.04573023 | 0.259994979 |
| DAG 34 :1 | 1.1 (0.99, 1.18) | 0.04648349 | 0.263944115 |
| TAG 52 :6 | 1.12 (1, 1.25) | 0.04718713 | 0.267601868 |
| DCER 22 :1 | 1.13 (1, 1.27) | 0.04861603 | 0.275531678 |
| TAG 47 :0 | 1.1 (0.99, 1.2) | 0.05000428 | 0.283221219 |
| LCER 18 :0 | 0.85 (0.72, 1) | 0.05123628 | 0.290016689 |
| TAG 49 :0 | 1.1 (0.99, 1.21) | 0.05524609 | 0.312124807 |
| LPC 22 :6 | 0.85 (0.72, 1) | 0.05660194 | 0.319383999 |
| TAG 56 :8 | 1.12 (0.99, 1.26) | 0.06083847 | 0.340090835 |
| DAG 34 :4 | 1.11 (0.99, 1.23) | 0.06158412 | 0.343760019 |
| TAG 44 :1 | 1.1 (0.99, 1.2) | 0.06367976 | 0.353340207 |
| LPE 16 :0 | 0.85 (0.7, 1.01) | 0.06432955 | 0.354823213 |
| CER 20 :0 | 1.12 (0.99, 1.27) | 0.06434128 | 0.354823213 |
| MAG 14 :1 | 1.09 (0.98, 1.19) | 0.06485479 | 0.356599646 |
| DAG 30 :2 | 1.07 (0.98, 1.14) | 0.06890434 | 0.365863776 |
| HCER 14 :0 | 0.87 (0.74, 1.01) | 0.07020666 | 0.370809802 |
| LPC 16 :0 | 0.85 (0.7, 1.01) | 0.07404787 | 0.386336725 |
| TAG 52 :7 | 1.1 (0.98, 1.2) | 0.07550309 | 0.392563736 |
| DCER 20 :0 | 1.12 (0.98, 1.27) | 0.08063171 | 0.413260452 |
| TAG 56 :7 | 1.11 (0.98, 1.24) | 0.08080071 | 0.413655528 |
| TAG 44 :2 | 1.08 (0.98, 1.18) | 0.08175154 | 0.415920768 |
| DAG 36 :3 | 1.1 (0.98, 1.22) | 0.08315034 | 0.421461711 |
| DAG 40 :8 | 1.11 (0.98, 1.25) | 0.08392097 | 0.42408126 |
| PI 36 :4 | 1.12 (0.98, 1.26) | 0.08593544 | 0.431595384 |
| TAG 48 :5 | 1.09 (0.98, 1.19) | 0.08647025 | 0.433237943 |
| DAG 40 :6 | 1.11 (0.98, 1.24) | 0.09083133 | 0.450413657 |
| DAG 36 :2 | 1.1 (0.97, 1.2) | 0.09133419 | 0.452149445 |
| TAG 53 :4 | 1.1 (0.98, 1.23) | 0.09348596 | 0.461026672 |
| DAG 36 :1 | 1.08 (0.97, 1.17) | 0.09785225 | 0.479406759 |
| PE 36 :2 | 1.11 (0.97, 1.26) | 0.1013339 | 0.494044119 |
| PC 34 :2 | 1.13 (0.97, 1.29) | 0.1036288 | 0.503323892 |
| DAG 38 :7 | 1.1 (0.97, 1.22) | 0.1047751 | 0.508537452 |
| TAG 55 :6 | 1.1 (0.97, 1.24) | 0.1082647 | 0.521406046 |
| SM 20 :1 | 0.89 (0.76, 1.03) | 0.1118745 | 0.536997719 |
| CE 18 :3 | 1.11 (0.97, 1.26) | 0.113383 | 0.542214014 |
| TAG 60 :11 | 1.11 (0.97, 1.27) | 0.1171271 | 0.558043165 |
| PC 32 :1 | 1.11 (0.97, 1.27) | 0.1173393 | 0.558758735 |
| TAG 57 :8 | 1.11 (0.97, 1.24) | 0.1180823 | 0.561597943 |
| TAG 54 :7 | 1.09 (0.97, 1.22) | 0.120614 | 0.571931649 |
| TAG 53 :7 | 1.1 (0.97, 1.23) | 0.1232599 | 0.583555582 |
| LCER 22 :0 | 0.89 (0.76, 1.03) | 0.1266614 | 0.598400339 |
| TAG 53 :3 | 1.09 (0.97, 1.22) | 0.1283186 | 0.604642592 |
| PE 34 :2 | 1.11 (0.97, 1.26) | 0.1330704 | 0.622147196 |
| TAG 51 :5 | 1.09 (0.97, 1.22) | 0.1342592 | 0.626728694 |
| DAG 33 :2 | 1.1 (0.96, 1.23) | 0.1378719 | 0.642593058 |
| TAG 54 :6 | 1.09 (0.96, 1.21) | 0.1386183 | 0.645403256 |
| CE 20 :5 | 1.09 (0.96, 1.22) | 0.1389149 | 0.646449833 |
| PE 40 :6 | 1.11 (0.96, 1.27) | 0.1403921 | 0.651303711 |
| PC 38 :5 | 0.9 (0.77, 1.04) | 0.1414716 | 0.655635458 |
| DAG 40 :5 | 1.09 (0.96, 1.2) | 0.1435421 | 0.66420529 |
| SM 16 :0 | 0.9 (0.78, 1.03) | 0.1439789 | 0.66588373 |
| DAG 40 :7 | 1.1 (0.96, 1.23) | 0.1441622 | 0.666159682 |
| LCER 20 :0 | 0.9 (0.78, 1.04) | 0.1441866 | 0.666159682 |
| TAG 56 :6 | 1.09 (0.96, 1.21) | 0.1446303 | 0.66718239 |
| TAG 52 :0 | 1.07 (0.96, 1.16) | 0.1454953 | 0.670485475 |
| SM 24 :1 | 0.9 (0.78, 1.04) | 0.1456714 | 0.670953381 |
| DAG 38 :2 | 1.08 (0.96, 1.18) | 0.146093 | 0.672550762 |
| TAG 51 :0 | 1.07 (0.96, 1.17) | 0.1485424 | 0.68277938 |
| LPE 18 :1 | 0.89 (0.75, 1.04) | 0.1496312 | 0.687432672 |
| CER 22 :1 | 1.1 (0.96, 1.24) | 0.1509123 | 0.692258296 |
| DAG 36 :5 | 1.09 (0.96, 1.21) | 0.1513918 | 0.693750627 |
| TAG 54 :8 | 1.09 (0.96, 1.21) | 0.1610858 | 0.733319396 |
| HCER 22 :1 | 0.9 (0.77, 1.04) | 0.1617823 | 0.73611775 |
| TAG 60 :12 | 1.1 (0.95, 1.25) | 0.1710952 | 0.742175461 |
| TAG 53 :2 | 1.08 (0.95, 1.19) | 0.1761158 | 0.742175461 |
| PE_P 36 :1 | 0.9 (0.78, 1.04) | 0.1789172 | 0.742175461 |
| TAG 54 :5 | 1.08 (0.96, 1.2) | 0.1808204 | 0.742175461 |
| TAG 56 :5 | 1.08 (0.95, 1.18) | 0.1808696 | 0.742175461 |
| SM 22 :0 | 1.09 (0.96, 1.24) | 0.1860128 | 0.742175461 |
| TAG 56 :9 | 1.09 (0.95, 1.23) | 0.1880785 | 0.742175461 |
| SM 20 :0 | 1.09 (0.95, 1.25) | 0.1982647 | 0.742175461 |
| HCER 18 :0 | 0.9 (0.77, 1.05) | 0.2035904 | 0.742175461 |
| PI 34 :1 | 1.09 (0.95, 1.24) | 0.2053289 | 0.742175461 |
| CE 18 :2 | 1.08 (0.96, 1.23) | 0.2061721 | 0.742175461 |
| TAG 46 :4 | 1.07 (0.95, 1.17) | 0.2063649 | 0.742175461 |
| PC 34 :0 | 1.09 (0.95, 1.25) | 0.2118243 | 0.742175461 |
| LPC 16 :1 | 0.9 (0.76, 1.05) | 0.2158166 | 0.742175461 |
| DAG 32 :0 | 1.07 (0.94, 1.18) | 0.2190959 | 0.742175461 |
| CE 16 :0 | 1.08 (0.95, 1.23) | 0.2230429 | 0.742175461 |
| TAG 60 :10 | 1.08 (0.94, 1.22) | 0.2307114 | 0.742175461 |
| PC 36 :5 | 1.09 (0.94, 1.24) | 0.2322691 | 0.742175461 |
| PI 36 :2 | 1.09 (0.94, 1.24) | 0.2328804 | 0.742175461 |
| TAG 54 :2 | 1.06 (0.94, 1.15) | 0.2334441 | 0.742175461 |
| TAG 42 :1 | 1.06 (0.95, 1.16) | 0.2372666 | 0.744696392 |
| TAG 44 :3 | 1.06 (0.95, 1.14) | 0.2411196 | 0.744696392 |
| CE 18 :4 | 1.08 (0.94, 1.21) | 0.2460046 | 0.747992159 |
| SM 18 :1 | 0.91 (0.78, 1.06) | 0.2477331 | 0.748684491 |
| SM 22 :1 | 0.92 (0.8, 1.06) | 0.2505813 | 0.751994573 |
| DAG 36 :4 | 1.06 (0.94, 1.16) | 0.253151 | 0.752240311 |
| TAG 54 :3 | 1.06 (0.94, 1.16) | 0.2550813 | 0.752700109 |
| CE 20 :2 | 1.08 (0.95, 1.22) | 0.2609359 | 0.76272255 |
| HCER 24 :0 | 0.92 (0.79, 1.07) | 0.271839 | 0.783397641 |
| TAG 54 :4 | 1.06 (0.94, 1.17) | 0.2740149 | 0.7873991 |
| DAG 38 :6 | 1.07 (0.94, 1.2) | 0.2747717 | 0.788316239 |
| PI 36 :1 | 1.08 (0.93, 1.23) | 0.2823072 | 0.802809871 |
| PC 35 :2 | 0.92 (0.77, 1.08) | 0.2892356 | 0.815559274 |
| PE_O 38 :4 | 0.92 (0.78, 1.07) | 0.2894045 | 0.815734451 |
| TAG 53 :1 | 1.06 (0.94, 1.15) | 0.2971928 | 0.828091461 |
| HCER 20 :0 | 0.92 (0.79, 1.07) | 0.2991654 | 0.831271629 |
| LPC 20 :3 | 0.92 (0.78, 1.07) | 0.3008035 | 0.834020693 |
| PE_O 36 :2 | 0.92 (0.8, 1.07) | 0.3017901 | 0.835983591 |
| PI 38 :5 | 0.93 (0.8, 1.07) | 0.3025851 | 0.837193538 |
| TAG 54 :1 | 1.05 (0.93, 1.14) | 0.3036494 | 0.839583549 |
| CE 15 :0 | 1.08 (0.93, 1.25) | 0.3124213 | 0.859396516 |
| PC 34 :4 | 1.07 (0.93, 1.23) | 0.3138924 | 0.861815628 |
| PE 34 :1 | 1.07 (0.93, 1.23) | 0.3200447 | 0.87443912 |
| CER 26 :0 | 1.07 (0.93, 1.23) | 0.3264636 | 0.888202087 |
| HCER 22 :0 | 0.93 (0.8, 1.08) | 0.3331893 | 0.901323719 |
| MAG 20 :0 | 0.93 (0.79, 1.06) | 0.3371745 | 0.910189056 |
| HCER 16 :0 | 0.93 (0.8, 1.08) | 0.3395484 | 0.91467691 |
| LPC 15 :0 | 0.92 (0.77, 1.09) | 0.3440532 | 0.922704463 |
| TAG 55 :8 | 1.06 (0.94, 1.2) | 0.3440662 | 0.922704463 |
| TAG 58 :10 | 1.06 (0.93, 1.19) | 0.3453681 | 0.925643971 |
| TAG 58 :7 | 1.06 (0.93, 1.17) | 0.3491837 | 0.931324352 |
| PE 36 :0 | 1.07 (0.92, 1.25) | 0.3561943 | 0.944370014 |
| DCER 16 :0 | 1.06 (0.93, 1.21) | 0.3672231 | 0.960478895 |
| SM 14 :0 | 1.07 (0.92, 1.23) | 0.3735871 | 0.964434707 |
| TAG 58 :9 | 1.06 (0.92, 1.19) | 0.3745647 | 0.964434707 |
| PC 30 :0 | 1.07 (0.92, 1.23) | 0.3776287 | 0.964434707 |
| TAG 58 :8 | 1.06 (0.92, 1.18) | 0.3826141 | 0.964434707 |
| TAG 54 :0 | 1.05 (0.92, 1.14) | 0.3835834 | 0.964434707 |
| LPE 20 :4 | 0.85 (0.57, 1.14) | 0.3848633 | 0.964434707 |
| LPE 22 :5 | 0.9 (0.71, 1.12) | 0.3905159 | 0.964434707 |
| MAG 16 :1 | 1.05 (0.92, 1.16) | 0.4026838 | 0.966260205 |
| SM 18 :0 | 1.06 (0.92, 1.21) | 0.4057973 | 0.967877146 |
| PC 36 :2 | 1.06 (0.92, 1.21) | 0.4128312 | 0.974680181 |
| TAG 56 :4 | 1.05 (0.92, 1.15) | 0.4168079 | 0.976772736 |
| PE_P 36 :2 | 0.94 (0.81, 1.09) | 0.4203481 | 0.978818459 |
| SM 26 :1 | 0.95 (0.83, 1.08) | 0.4234832 | 0.982812968 |
| PE_O 38 :5 | 0.94 (0.8, 1.09) | 0.4307752 | 0.992569571 |
| TAG 58 :6 | 1.04 (0.91, 1.14) | 0.435705 | 0.99995621 |
| PC 33 :2 | 1.07 (0.91, 1.26) | 0.437524 | 0.99995621 |
| PI 38 :3 | 1.05 (0.92, 1.21) | 0.4444401 | 0.99995621 |
| SM 24 :0 | 1.05 (0.92, 1.18) | 0.4560726 | 0.99995621 |
| PE_P 34 :1 | 0.95 (0.81, 1.09) | 0.4560957 | 0.99995621 |
| CER 24 :1 | 1.05 (0.92, 1.2) | 0.4585526 | 0.99995621 |
| PE 38 :4 | 1.05 (0.92, 1.19) | 0.4652344 | 0.99995621 |
| PC 40 :5 | 1.05 (0.91, 1.21) | 0.4666304 | 0.99995621 |
| TAG 55 :5 | 1.04 (0.92, 1.16) | 0.4682364 | 0.99995621 |
| PC 36 :0 | 1.05 (0.91, 1.21) | 0.479712 | 0.99995621 |
| PE_P 38 :4 | 0.95 (0.8, 1.1) | 0.4832535 | 0.99995621 |
| PE 38 :6 | 1.05 (0.9, 1.22) | 0.4889982 | 0.99995621 |
| CER 20 :1 | 1.05 (0.91, 1.2) | 0.4912296 | 0.99995621 |
| PE_P 40 :6 | 0.95 (0.81, 1.1) | 0.4955428 | 0.99995621 |
| HCER 18 :1 | 0.95 (0.82, 1.1) | 0.4955754 | 0.99995621 |
| PE_P 38 :6 | 1.05 (0.9, 1.22) | 0.501852 | 0.99995621 |
| PC 40 :6 | 1.05 (0.91, 1.21) | 0.5078366 | 0.99995621 |
| TAG 57 :2 | 1.04 (0.91, 1.17) | 0.5150881 | 0.99995621 |
| DAG 38 :5 | 1.04 (0.91, 1.16) | 0.5189965 | 0.99995621 |
| PE 40 :5 | 1.04 (0.91, 1.18) | 0.5214777 | 0.99995621 |
| PE_P 40 :4 | 0.95 (0.82, 1.1) | 0.523451 | 0.99995621 |
| CE 18 :0 | 1.04 (0.91, 1.18) | 0.525382 | 0.99995621 |
| TAG 58 :3 | 0.95 (0.79, 1.08) | 0.5264827 | 0.99995621 |
| PC 36 :3 | 1.05 (0.9, 1.21) | 0.5320746 | 0.99995621 |
| PE_O 36 :4 | 0.95 (0.81, 1.11) | 0.533628 | 0.99995621 |
| DAG 38 :3 | 1.04 (0.91, 1.16) | 0.5367403 | 0.99995621 |
| MAG 18 :2 | 1.03 (0.89, 1.13) | 0.5551246 | 0.99995621 |
| PC 38 :2 | 1.04 (0.9, 1.2) | 0.5615081 | 0.99995621 |
| PE 40 :4 | 1.04 (0.9, 1.2) | 0.5813862 | 0.99995621 |
| PC 34 :1 | 1.04 (0.9, 1.2) | 0.5816934 | 0.99995621 |
| CE 22 :6 | 1.04 (0.9, 1.2) | 0.5876576 | 0.99995621 |
| PE_P 34 :0 | 0.96 (0.84, 1.1) | 0.5884995 | 0.99995621 |
| TAG 56 :2 | 1.03 (0.9, 1.13) | 0.591536 | 0.99995621 |
| PE 34 :3 | 0.97 (0.85, 1.1) | 0.6055621 | 0.99995621 |
| PE_P 40 :5 | 0.96 (0.82, 1.11) | 0.605577 | 0.99995621 |
| PE_P 38 :5 | 0.96 (0.81, 1.12) | 0.6107304 | 0.99995621 |
| TAG 56 :3 | 1.03 (0.89, 1.13) | 0.6192626 | 0.99995621 |
| CE 22 :4 | 0.97 (0.84, 1.11) | 0.6261547 | 0.99995621 |
| DAG 34 :0 | 1.03 (0.88, 1.15) | 0.6361138 | 0.99995621 |
| CE 24 :0 | 0.97 (0.84, 1.08) | 0.6422535 | 0.99995621 |
| MAG 18 :1 | 0.95 (0.72, 1.1) | 0.6455748 | 0.99995621 |
| TAG 55 :4 | 1.03 (0.9, 1.13) | 0.6458518 | 0.99995621 |
| TAG 58 :5 | 1.03 (0.88, 1.12) | 0.6608776 | 0.99995621 |
| TAG 57 :3 | 1.03 (0.89, 1.15) | 0.6661289 | 0.99995621 |
| PI 38 :4 | 1.03 (0.9, 1.17) | 0.670464 | 0.99995621 |
| PE_O 36 :1 | 0.97 (0.84, 1.11) | 0.6726625 | 0.99995621 |
| PC 34 :3 | 1.03 (0.88, 1.2) | 0.6748435 | 0.99995621 |
| CE 24 :1 | 0.98 (0.84, 1.08) | 0.6820945 | 0.99995621 |
| CE 20 :4 | 0.97 (0.85, 1.11) | 0.6884354 | 0.99995621 |
| PC 38 :4 | 0.97 (0.84, 1.12) | 0.6909784 | 0.99995621 |
| PC 32 :0 | 1.03 (0.89, 1.19) | 0.6911358 | 0.99995621 |
| PE 36 :1 | 1.03 (0.88, 1.18) | 0.7133128 | 0.99995621 |
| TAG 55 :3 | 1.02 (0.89, 1.12) | 0.7167384 | 0.99995621 |
| MAG 18 :3 | 1.02 (0.89, 1.14) | 0.7200814 | 0.99995621 |
| TAG 55 :2 | 1.02 (0.89, 1.12) | 0.7226261 | 0.99995621 |
| CER 16 :0 | 1.02 (0.89, 1.17) | 0.7308723 | 0.99995621 |
| TAG 56 :10 | 1.02 (0.9, 1.12) | 0.7321373 | 0.99995621 |
| PE 36 :4 | 1.02 (0.89, 1.17) | 0.7404617 | 0.99995621 |
| PE_P 36 :3 | 1.02 (0.89, 1.17) | 0.7576401 | 0.99995621 |
| LPE 20 :3 | 0.97 (0.77, 1.2) | 0.769301 | 0.99995621 |
| CER 14 :0 | 0.98 (0.84, 1.14) | 0.7953447 | 0.99995621 |
| PE 38 :5 | 0.98 (0.85, 1.13) | 0.8104041 | 0.99995621 |
| PI 36 :3 | 1.02 (0.88, 1.17) | 0.8221099 | 0.99995621 |
| CE 12 :0 | 0.98 (0.82, 1.14) | 0.8298989 | 0.99995621 |
| CE 22 :0 | 1.01 (0.89, 1.15) | 0.831167 | 0.99995621 |
| CE 17 :0 | 1.01 (0.88, 1.16) | 0.8522139 | 0.99995621 |
| PE 38 :2 | 0.99 (0.85, 1.14) | 0.8567909 | 0.99995621 |
| PC 36 :1 | 0.99 (0.85, 1.14) | 0.8604518 | 0.99995621 |
| DAG 38 :4 | 0.99 (0.86, 1.12) | 0.8706604 | 0.99995621 |
| PE_P 38 :3 | 1.01 (0.88, 1.16) | 0.8822804 | 0.99995621 |
| CER 26 :1 | 0.99 (0.86, 1.14) | 0.889231 | 0.99995621 |
| CE 22 :5 | 1.01 (0.88, 1.16) | 0.897226 | 0.99995621 |
| CE 22 :1 | 1.01 (0.9, 1.11) | 0.8991586 | 0.99995621 |
| LPE 18 :2 | 0.99 (0.84, 1.15) | 0.9179414 | 0.99995621 |
| CE 22 :2 | 1.01 (0.89, 1.11) | 0.9224974 | 0.99995621 |
| TAG 56 :1 | 1.01 (0.88, 1.11) | 0.9307919 | 0.99995621 |
| PE_P 40 :7 | 0.99 (0.85, 1.16) | 0.9385893 | 0.99995621 |
| PE_P 36 :4 | 0.99 (0.85, 1.16) | 0.9461458 | 0.99995621 |
| PC 36 :4 | 1 (0.87, 1.15) | 0.9557177 | 0.99995621 |
| SM 26 :0 | 1 (0.89, 1.12) | 0.9582735 | 0.99995621 |
| CE 20 :0 | 1 (0.89, 1.1) | 0.9664192 | 0.99995621 |
| PC 38 :6 | 1 (0.85, 1.16) | 0.9666557 | 0.99995621 |
| PC 40 :4 | 1 (0.86, 1.15) | 0.970256 | 0.99995621 |
| LPE 22 :6 | 1 (0.81, 1.15) | 0.9728619 | 0.99995621 |
| PE 36 :3 | 1 (0.87, 1.14) | 0.9811359 | 0.99995621 |
| PE_P 34 :2 | 1 (0.87, 1.15) | 0.9826827 | 0.99995621 |
| CE 20 :1 | 1 (0.89, 1.1) | 0.9826936 | 0.99995621 |
| CER 18 :1 | 1 (0.87, 1.14) | 0.9844107 | 0.99995621 |
| CE 18 :1 | 1 (0.87, 1.14) | 0.9847647 | 0.99995621 |

^1^Individual Poisson log-risk regression models adjusted for race, CARDIA center, age, education, sex, smoking, dietary intake (food groups, total energy, APDQS), eGFR, BMI, hypertension, and lipid-lowering medication use (Y20 variables). ^2^

^2^For lipids containing >1 fatty acyl the lipid species is the total sum of carbon atoms and double bonds present in the lipid.

^3^P-values were adjusted for the FDR using the Benjamin-Hochberg procedure.

Supplemental figure 2A-B. Minimally-adjusted associations^1,2^ between lipid species^2^ and MASLD. A. Adjusted for sociodemographics, and B. additionally adjusted for health behaviors.


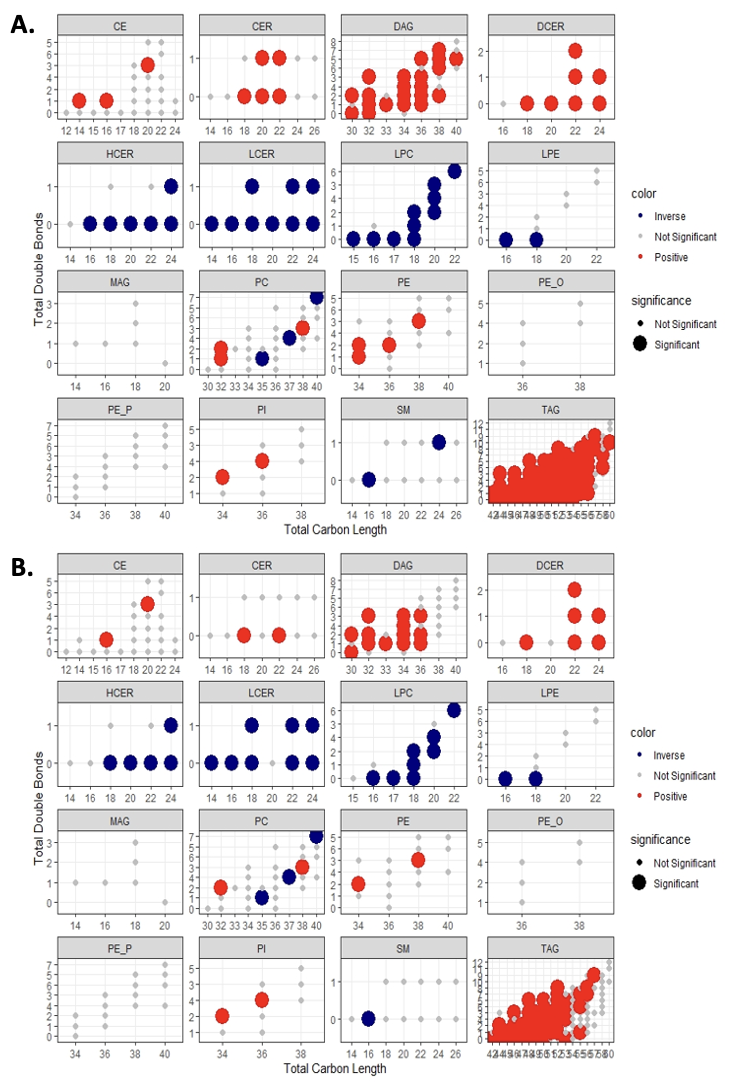


^1^Individual Poisson log-risk regression models adjusted for: M1 = race, CARDIA center, age, education, and sex; M2 = M1 + smoking, dietary intake (food groups, total energy, APDQS).

^2^P-values were adjusted for the FDR using the Benjamin-Hochberg procedure. Significant lipids are those with an adjusted p-value <0.05.

Supplemental figure 3A-B. Multivariable-adjusted associations^1,2^ between TAG species^3^ and MASLD A) unadjusted for total TAGs, and B) adjusted for total TAGs.


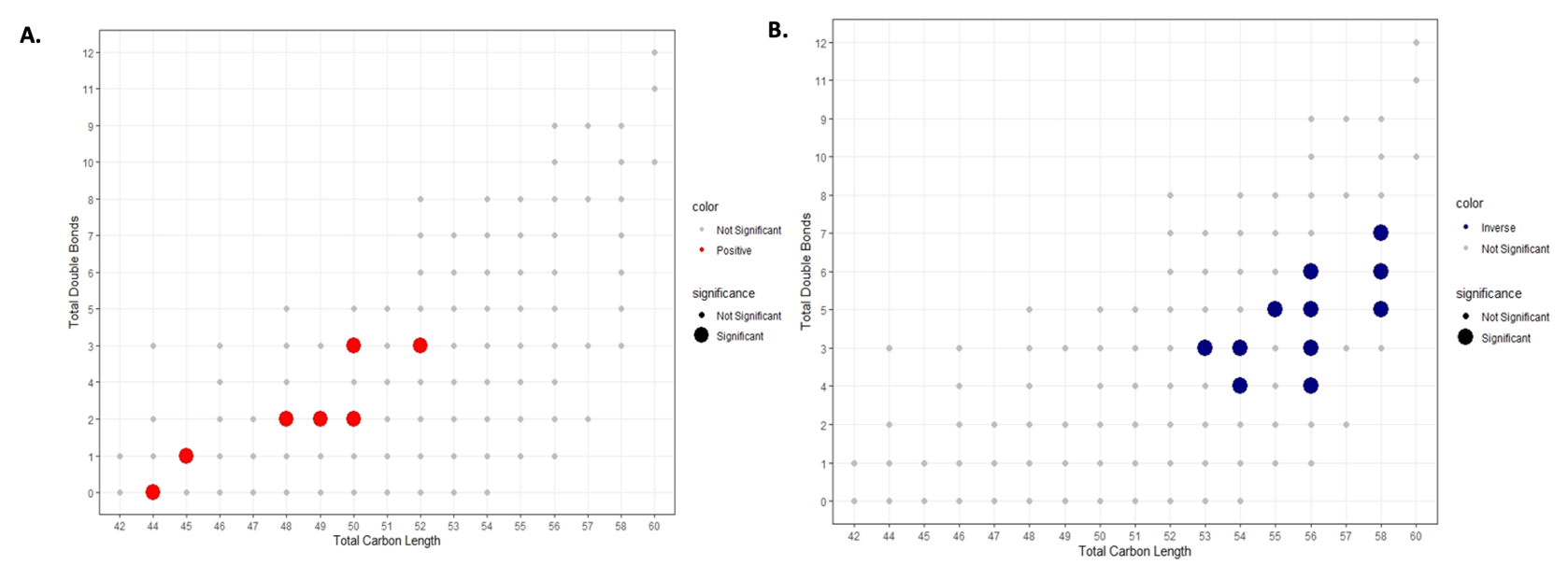


^1^Multivariable-adjusted risk ratios (95% CI) from a Poisson regression model adjusted for: A. race (Black/White), examination center (Birmingham, AL; Chicago, IL; ), age (continuous), attained education (continuous), sex (male/female), smoking (current/not current), physical activity score (continuous), dietary intake as food groups (continuous), caloric intake (continuous), a priori diet quality score (continuous), eGFR (continuous), BMI (continuous), hypertension (yes/no), and lipid-lowering cholesterol medication use (yes/no), and B: A + total TAGs.

^2^P-values were adjusted for the FDR using the Benjamin-Hochberg procedure. Significant lipids are those with an adjusted p-value <0.05.

Supplemental figure 4. Lambda parameter tuning of penalized (LASSO) logistic regression model^1^ of molecular lipid species^2^ and MASLD.


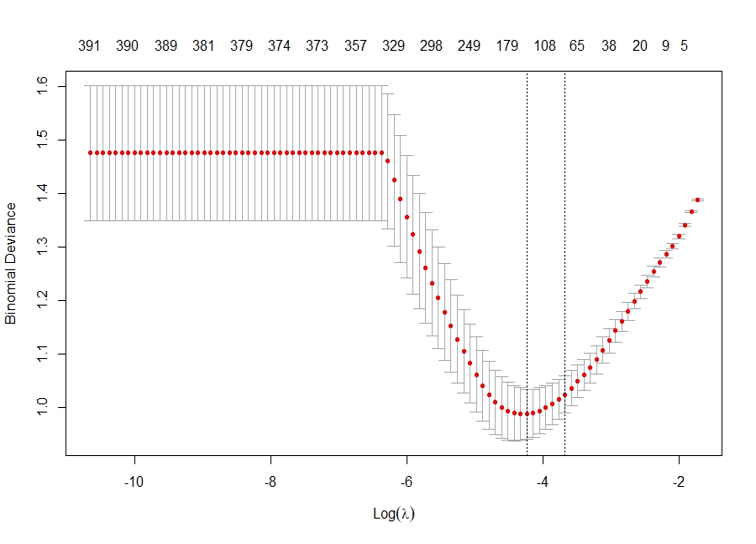


^1^Logistic regression model with a LASSO penalty trained on a random case-oversampled 0.7 split and tested on the remaining 0.3

^2^Sensitivity (95% CI) = 68% (56%-79%); specificity = 71% (65%-76%)

^3^Molecular lipid species (n=756)

Supplemental table 4. Coefficients for selected molecular lipid species^1^ from penalized (LASSO) regression model^2^ used in weighted MASLD-LRS

| LASSO-Selected Molecular Lipid Specie | Lipid Class | LRS weight^3^ |
| --- | --- | --- |
| TAG44_1_FA16_1_uM | TAG | 0.23087445 |
| TAG49_1_FA16_1_uM | TAG | 0.19621998 |
| HCER_24_1_uM | HCE | 0.19005291 |
| PE_P_16_0_20_3_uM | PE | 0.1627765 |
| PE_18_0_22_5_uM | PE | 0.16102876 |
| PE_18_0_18_2_uM | PE | 0.1589147 |
| CER_22_1_uM | CER | 0.15485437 |
| PC_18_1_22_6_uM | PC | 0.14681159 |
| TAG42_1_FA16_0_uM | TAG | 0.14181872 |
| DCER_22_0_uM | DCE | 0.14142857 |
| TAG46_1_FA18_0_uM | TAG | 0.13903369 |
| TAG53_6_FA18_2_uM | TAG | 0.13721031 |
| TAG53_3_FA18_0_uM | TAG | 0.13274997 |
| CE_22_2_uM | CE | 0.12541259 |
| TAG54_6_FA18_3_uM | TAG | 0.12506118 |
| PC_18_2_16_1_uM | PC | 0.12201882 |
| TAG46_1_FA14_1_uM | TAG | 0.11933835 |
| TAG60_10_FA22_5_uM | TAG | 0.11911109 |
| PC_20_0_18_2_uM | PC | 0.109855 |
| TAG55_6_FA20_3_uM | TAG | 0.10948368 |
| TAG52_2_FA18_2_uM | TAG | 0.10812666 |
| DAG_16_0_18_0_uM | DAG | 0.10747902 |
| PE_18_0_20_4_uM | PE | 0.10695611 |
| LCER_16_0_uM | LCE | 0.10632103 |
| TAG50_2_FA20_2_uM | TAG | 0.1030067 |
| TAG44_0_FA14_0_uM | TAG | 0.09778482 |
| TAG58_7_FA16_0_uM | TAG | 0.09473624 |
| DAG_18_1_20_4_uM | DAG | 0.09460128 |
| PE_O_18_0_18_1_uM | PE | 0.09451076 |
| CE_22_0_uM | CE | 0.08946859 |
| TAG56_5_FA18_0_uM | TAG | 0.08766245 |
| TAG52_3_FA18_0_uM | TAG | 0.08341293 |
| DCER_24_0_uM | DCE | 0.08171936 |
| DCER_22_2_uM | DCE | 0.07804275 |
| LPC_16_0_uM | LPC | 0.07730449 |
| DAG_18_1_20_2_uM | DAG | 0.07574291 |
| PE_P_16_0_20_4_uM | PE | 0.07549798 |
| PC_18_0_18_0_uM | PC | 0.07441565 |
| TAG58_7_FA20_4_uM | TAG | 0.07134629 |
| PE_18_0_22_6_uM | PE | 0.07093245 |
| LCER_18_1_uM | LCE | 0.06810585 |
| TAG54_7_FA20_5_uM | TAG | 0.0679171 |
| LPC_18_2_uM | LPC | 0.06639005 |
| PC_18_0_18_1_uM | PC | 0.06628852 |
| LPC_20_4_uM | LPC | 0.06444234 |
| TAG48_4_FA18_1_uM | TAG | 0.06292948 |
| CE_22_1_uM | CE | 0.06281395 |
| TAG54_4_FA20_3_uM | TAG | 0.06043323 |
| LCER_14_0_uM | LCE | 0.06040554 |
| PC_14_0_18_1_uM | PC | 0.06018264 |
| TAG50_5_FA16_0_uM | TAG | 0.05918212 |
| TAG56_7_FA18_0_uM | TAG | 0.05904393 |
| TAG54_8_FA18_3_uM | TAG | 0.05719969 |
| TAG56_6_FA22_5_uM | TAG | 0.05696706 |
| TAG54_4_FA22_4_uM | TAG | 0.05645309 |
| TAG55_6_FA18_1_uM | TAG | 0.05585239 |
| CER_18_0_uM | CER | 0.05557956 |
| CE_12_0_uM | CE | 0.05503426 |
| TAG54_5_FA22_4_uM | TAG | 0.05463265 |
| PE_O_16_0_20_4_uM | PE | 0.05454065 |
| TAG52_6_FA18_3_uM | TAG | 0.05434103 |
| PE_P_18_0_18_1_uM | PE | 0.05406914 |
| TAG53_7_FA18_3_uM | TAG | 0.05368536 |
| TAG50_1_FA20_1_uM | TAG | 0.05359872 |
| PC_17_0_20_4_uM | PC | 0.05302246 |
| TAG52_2_FA18_0_uM | TAG | 0.0524764 |
| PC_18_0_22_4_uM | PC | 0.05221997 |
| DAG_14_0_20_0_uM | DAG | 0.05164398 |
| TAG52_4_FA22_1_uM | TAG | 0.05120515 |
| PC_18_0_20_3_uM | PC | 0.05109234 |
| TAG56_2_FA18_0_uM | TAG | 0.04988152 |
| CE_17_0_uM | CE | 0.04871258 |
| PC_18_1_20_5_uM | PC | 0.04799007 |
| TAG49_2_FA14_0_uM | TAG | 0.04580363 |
| DCER_22_1_uM | DCE | 0.04576828 |
| LPE_18_0_uM | LPE | 0.0455935 |
| PE_16_0_20_4_uM | PE | 0.04330022 |
| CE_20_1_uM | CE | 0.04321324 |
| TAG50_2_FA18_0_uM | TAG | 0.04312825 |
| TAG56_4_FA18_2_uM | TAG | 0.04221235 |
| DCER_24_1_uM | DCE | 0.04213125 |
| HCER_22_1_uM | HCE | 0.04138286 |
| TAG56_6_FA20_2_uM | TAG | 0.0410225 |
| TAG48_5_FA18_3_uM | TAG | 0.04068721 |
| PI_16_0_18_2_uM | PI | 0.04010237 |
| PC_18_1_22_5_uM | PC | 0.0379107 |
| TAG58_7_FA18_0_uM | TAG | 0.03487182 |
| PC_14_0_20_4_uM | PC | 0.03472554 |
| TAG56_8_FA22_5_uM | TAG | 0.03444888 |
| TAG48_0_FA14_0_uM | TAG | 0.03422629 |
| CE_16_1_uM | CE | 0.03366083 |
| TAG47_2_FA15_0_uM | TAG | 0.03274797 |
| TAG54_6_FA18_1_uM | TAG | 0.03238333 |
| LCER_24_1_uM | LCE | 0.03218634 |
| TAG57_9_FA22_6_uM | TAG | 0.03094472 |
| TAG53_3_FA18_2_uM | TAG | 0.02966556 |
| PI_18_1_18_1_uM | PI | 0.02952554 |
| LCER_22_1_uM | LCE | 0.02929252 |
| TAG49_1_FA14_0_uM | TAG | 0.02912246 |
| HCER_22_0_uM | HCE | 0.02802197 |
| PC_16_0_22_4_uM | PC | 0.02687697 |
| TAG50_2_FA18_1_uM | TAG | 0.02638331 |
| TAG55_3_FA18_2_uM | TAG | 0.02616495 |
| TAG51_0_FA17_0_uM | TAG | 0.0253718 |
| TAG53_5_FA18_2_uM | TAG | 0.02535232 |
| PE_P_16_0_22_6_uM | PE | 0.02491798 |
| TAG53_5_FA18_3_uM | TAG | 0.02482409 |
| CER_20_1_uM | CER | 0.02463644 |
| DAG_16_1_18_3_uM | DAG | 0.0222235 |
| TAG56_8_FA22_6_uM | TAG | 0.02086865 |
| LPC_20_2_uM | LPC | 0.0205569 |
| TAG56_5_FA20_2_uM | TAG | 0.02041338 |
| DAG_14_0_16_1_uM | DAG | 0.01975091 |
| TAG51_2_FA16_1_uM | TAG | 0.01964395 |
| TAG50_5_FA18_3_uM | TAG | 0.01958136 |
| TAG53_1_FA16_0_uM | TAG | 0.01951446 |
| TAG52_8_FA16_1_uM | TAG | 0.01939005 |
| TAG54_5_FA20_2_uM | TAG | 0.01935667 |
| DCER_20_0_uM | DCE | 0.01913784 |
| TAG46_3_FA12_0_uM | TAG | 0.01892494 |
| TAG49_3_FA16_0_uM | TAG | 0.01854984 |
| LPC_20_3_uM | LPC | 0.01771257 |
| CE_22_4_uM | CE | 0.01756499 |
| DAG_18_0_22_6_uM | DAG | 0.01680284 |
| PC_16_0_20_4_uM | PC | 0.01433568 |
| TAG50_3_FA14_1_uM | TAG | 0.01423054 |
| SM_14_0_uM | SM | 0.01414388 |
| TAG44_1_FA12_0_uM | TAG | 0.01392314 |
| LPC_16_1_uM | LPC | 0.01363688 |
| SM_26_0_uM | SM | 0.01325552 |
| TAG58_6_FA18_1_uM | TAG | 0.01276313 |
| TAG52_3_FA18_2_uM | TAG | 0.01206676 |
| DAG_18_0_18_3_uM | DAG | 0.01059286 |
| TAG58_9_FA18_2_uM | TAG | 0.01000318 |
| TAG54_6_FA20_4_uM | TAG | 0.00971701 |
| MAG_18_2_uM | MAG | 0.00926415 |
| TAG44_0_FA18_0_uM | TAG | 0.0088553 |
| CE_20_4_uM | CE | 0.00878417 |
| DAG_16_0_20_5_uM | DAG | 0.00842398 |
| HCER_24_0_uM | HCE | 0.00812823 |
| CER_22_0_uM | CER | 0.00789257 |
| TAG54_3_FA18_2_uM | TAG | 0.00783577 |
| TAG52_6_FA18_1_uM | TAG | 0.00737196 |
| TAG51_0_FA18_0_uM | TAG | 0.00691445 |
| TAG52_4_FA20_2_uM | TAG | 0.0065567 |
| PE_P_18_0_16_0_uM | PE | 0.00613472 |
| TAG54_4_FA16_1_uM | TAG | 0.00609038 |
| PC_16_0_20_1_uM | PC | 0.00508374 |
| CE_18_0_uM | CE | 0.00503005 |
| TAG56_7_FA18_2_uM | TAG | 0.00412766 |
| PE_18_0_18_0_uM | PE | 0.00358279 |
| PC_18_1_20_3_uM | PC | 0.00333567 |
| TAG56_3_FA16_0_uM | TAG | 0.00308044 |
| TAG46_1_FA12_0_uM | TAG | 0.00305349 |
| TAG52_4_FA16_0_uM | TAG | 0.0025297 |
| MAG_18_3_uM | MAG | 0.00173698 |
| PI_18_1_18_2_uM | PI | 0.00112221 |
| TAG51_1_FA16_0_uM | TAG | 0.00096179 |
| TAG52_8_FA18_2_uM | TAG | 0.00029065 |

^1^Molecular lipid species (n=756)

^2^Penalized (LASSO) regression model trained on a random case-oversampled 0.7 subset, and tested on the remaining 0.3

^3^Beta coefficient from penalized (LASSO) regression model; |coefficient| used in MASLD-LRS equation.

Supplemental figure 5. Correlations between choline metabolites and lipid classes


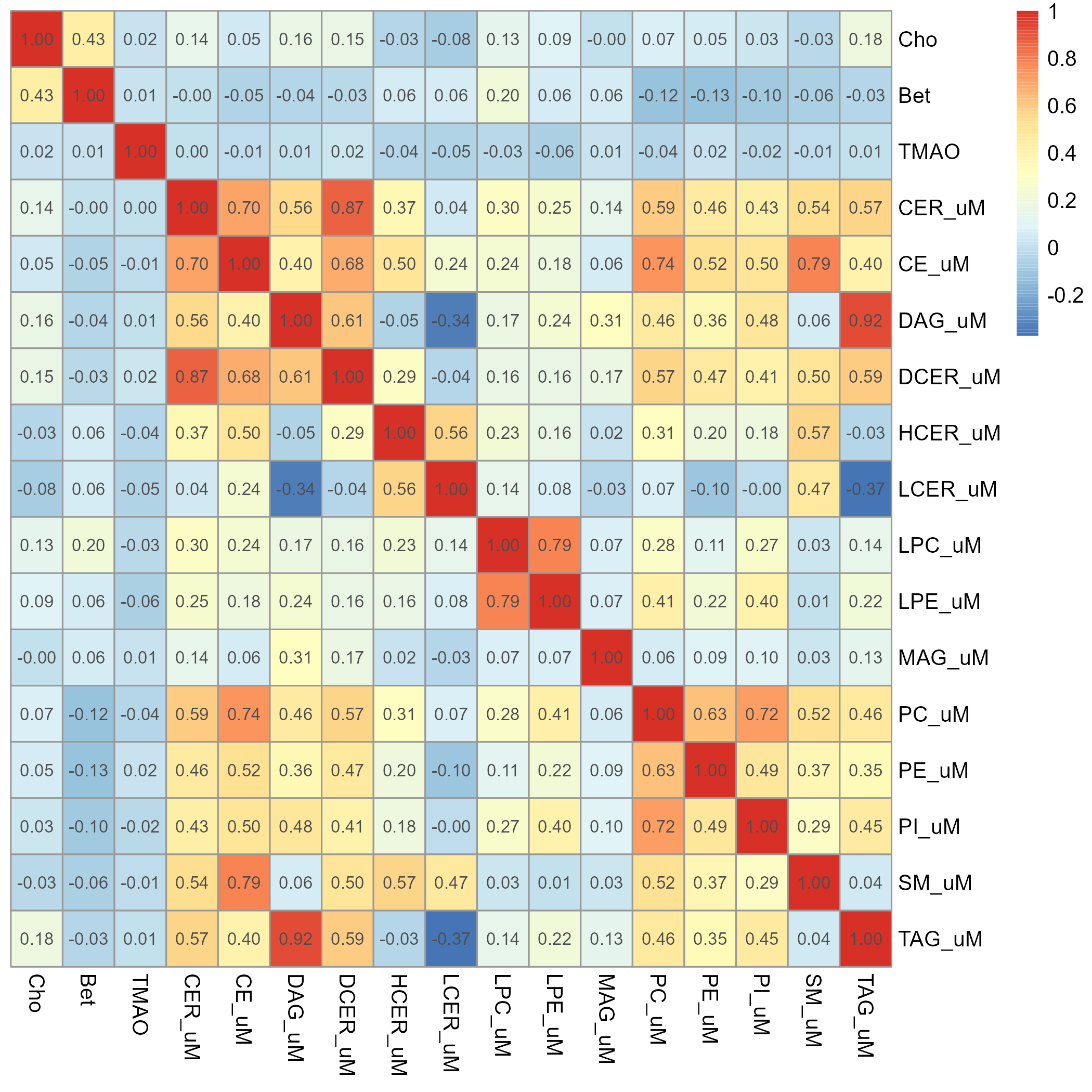


Supplemental figure 6. Multivariable-adjusted associations^1^ between choline metabolites and lipid classes.


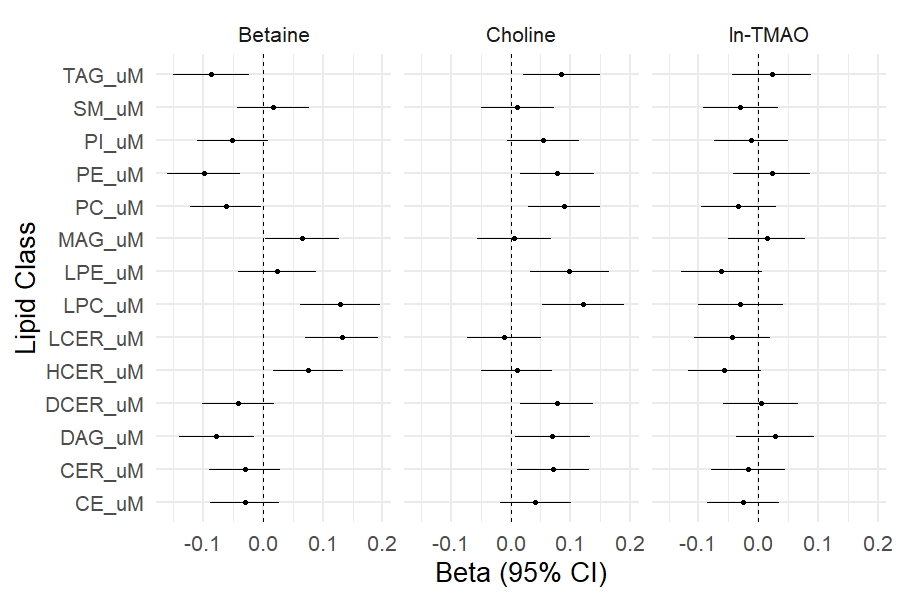


^1^Individual linear regression models adjusted for race, CARDIA examination center, age, sex, education, smoking, physical activity, diet (food groups, total energy, APDQS), eGFR, BMI, hypertension, and lipid-lowering medication use ; n=1,022; 94 observations deleted due to missingness.

Supplemental table 5. Descriptive characteristics of the study population stratified by tertiles of the LASSO derived MASLD-LRS

|  |  | MASLD-LRS Tertile | | |  |
| --- | --- | --- | --- | --- | --- |
| Characteristic^1^ | Overall | 1 | 2 | 3 | p-value^2^ |
| N | 1,116 | 372 | 372 | 372 |  |
| Race, % White | 57 | 57 | 52 | 61 | 0.06 |
| CARDIA Center, % |  |  |  |  | 0.43 |
| 1 | 28 | 31 | 24 | 30 |  |
| 2 | 18 | 18 | 20 | 16 |  |
| 3 | 31 | 30 | 31 | 33 |  |
| 4 | 22 | 22 | 24 | 21 |  |
| Age, yrs. | 26 (22, 28) | 26 (23, 28) | 26 (22, 28) | 26 (22, 28) | 0.62 |
| Attained education, yrs. | 16 (14, 18) | 16 (14, 18) | 16 (14, 18) | 16 (14, 18) | 0.02 |
| Sex, % female | 58 | 67 | 61 | 46 | <0.01 |
| Physical activity score, med (IQR) | 276 (130, 490) | 280 (144, 487) | 286 (127, 532) | 258 (120, 468) | 0.55 |
| Current smoking, % yes | 15 | 12 | 14 | 18 | 0.11 |
| eGFR | 96 (83, 104) | 93 (83, 103) | 97 (83, 108) | 95 (84, 104) | 0.36 |
| BMI (kg/m2) | 28 (25, 33) | 26 (23, 29) | 28 (25, 32) | 31 (27, 35) | <0.01 |
| Hypertension, % yes | 20 | 15 | 16 | 29 | <0.01 |
| Lipid-lowering medication use, % yes | 8.7 | 6.5 | 6.7 | 13 | <0.01 |
| APDQS | 62 (54, 70) | 64 (56, 73) | 61 (54, 70) | 62 (54, 68) | <0.01 |

^1^Median (IQR) unless otherwise noted

^2^Pearson’s Chi-squared test; Kruskal-Wallis rank sum test

Supplemental figure 7. Multivariable-adjusted^1^ associations^2^ between choline metabolites and the MASLD-LRS


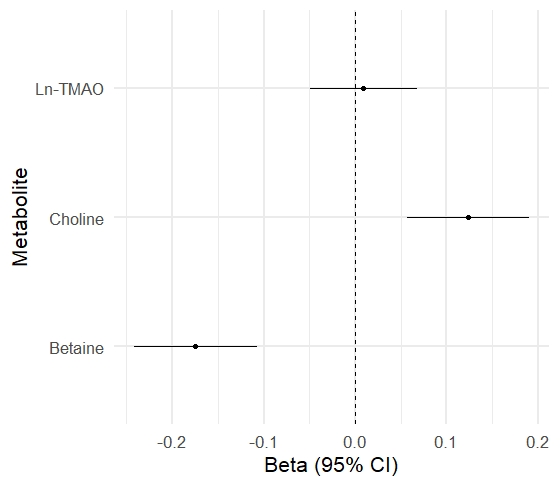


^1^Linear regression model adjusted for race, CARDIA examination center, age, sex, education, smoking, physical activity, diet (food groups, total energy, APDQS), eGFR, BMI, hypertension, lipid-lowering medication use, and the other two choline metabolites.; n=1,022; 94 observations deleted due to missingness.

^2^Beta (95% CI): choline = 0.12 (0.06, 0.19), betaine = -0.17 (-0.24, -0.11), ln-TMAO = 0.01 (-0.05, 0.07).
